# Supplementary material for: Discovery Stack Pilot demonstrates the feasibility and outcomes of a scientist-designed peer-review model that separates quality and impact
Source: PLoS Biol. 2026 Sep 8;24(9):e3003986. doi: 10.1371/journal.pbio.3003986 (PMC13614658; doi:10.1371/journal.pbio.3003986)
Supplement: Supplementary file 1 [file pbio.3003986.s009.docx]

| **Figure** | **n_**  **NEW** | **n_**  **DSP** | **Median**  **NEW** | **Median**  **DSP** | **Mean**  **NEW** | **Mean**  **DSP** | **Mean**  **Diff** | **95% CI**  **Lower** | **95%**  **CI**  **Upper** | **Mann-Whitney U** | **p-value (raw)** | **p-value (BH)** | **Effect size (r)** | **Survey**  **#** |
| --- | --- | --- | --- | --- | --- | --- | --- | --- | --- | --- | --- | --- | --- | --- |
| Fig. 7A, Row 1 | 46 | 39 | 2 | 2 | 1.93 | 1.54 | 0.40 | 0.11 | 0.69 | 1125.5 | 0.024 | 0.194 | 0.25 | DSPQ1 |
| Fig. 7A, Row 2 | 46 | 39 | 2 | 2 | 2.48 | 1.82 | 0.66 | 0.36 | 0.96 | 1284.5 | 0.0002 | 0.005 | 0.41 | DSPQ2 |
| Fig. 3A | 47 | 39 | 2 | 1 | 1.91 | 1.62 | 0.30 | -0.04 | 0.66 | 1065.5 | 0.163 | 0.538 | 0.15 | Q1 |
| Fig. 6J | 23 | 19 | 2 | 2 | 2.57 | 2.26 | 0.30 | -0.21 | 0.83 | 249.5 | 0.408 | 0.773 | 0.13 | Q11+O7 |
| Fig. 4C, Row 5 | 21 | 19 | 2 | 2 | 2.29 | 2.26 | 0.02 | -0.56 | 0.58 | 193.5 | 0.868 | 0.946 | -0.03 | Q12 |
| Fig. 4A, Row 2 | 21 | 19 | 2 | 2 | 2.14 | 2.26 | -0.12 | -0.88 | 0.65 | 181 | 0.611 | 0.823 | -0.08 | Q12b |
| Fig. 4C, Row 1 | 23 | 19 | 1 | 2 | 1.52 | 1.68 | -0.16 | -0.61 | 0.27 | 201.5 | 0.640 | 0.823 | -0.07 | Q13 |
| Fig. 4C, Row 2 | 23 | 19 | 1 | 2 | 1.65 | 1.74 | -0.08 | -0.58 | 0.42 | 206.5 | 0.750 | 0.920 | -0.05 | Q14 |
| Fig. 4C, Row 6 | 23 | 19 | 2 | 1 | 2.00 | 1.89 | 0.11 | -0.48 | 0.67 | 240.5 | 0.564 | 0.801 | 0.09 | Q15 |
| Fig. 4A, Row 1 | 21 | 20 | 2 | 2 | 2.05 | 1.90 | 0.15 | -0.33 | 0.63 | 232 | 0.551 | 0.801 | 0.09 | Q15b |
| Fig. 4C, Row 7 | 23 | 19 | 2 | 3 | 2.17 | 2.37 | -0.19 | -0.83 | 0.48 | 188 | 0.431 | 0.773 | -0.12 | Q16 |
| Fig. 4C, Row 4 | 23 | 19 | 2 | 2 | 2.00 | 2.05 | -0.05 | -0.54 | 0.44 | 215 | 0.936 | 0.946 | -0.01 | Q17 |
| Fig. 4C, Row 3 | 23 | 19 | 2 | 2 | 2.04 | 1.84 | 0.20 | -0.30 | 0.72 | 245 | 0.486 | 0.773 | 0.11 | Q18 |
| Fig. 2H | 47 | 39 | 2 | 2 | 1.94 | 1.59 | 0.35 | 0.01 | 0.67 | 1078 | 0.129 | 0.538 | 0.16 | Q2 |
| Fig. 2I | 47 | 39 | 2 | 2 | 1.87 | 1.74 | 0.13 | -0.19 | 0.45 | 992 | 0.463 | 0.773 | 0.08 | Q21 |
| Fig. 5C, Row 1 | 40 | 35 | 2 | 2 | 2.00 | 2.00 | 0.00 | -0.43 | 0.43 | 693.5 | 0.946 | 0.946 | -0.01 | Q24 |
| Fig. 5C, Row 2 | 40 | 35 | 2 | 2 | 1.78 | 1.63 | 0.15 | -0.15 | 0.45 | 761 | 0.467 | 0.773 | 0.08 | Q25 |
| Fig. 5C, Row 3 | 39 | 35 | 2 | 2 | 1.97 | 1.94 | 0.03 | -0.25 | 0.31 | 698.5 | 0.837 | 0.946 | 0.02 | Q26 |
| Fig. 5C, Row 4 | 50 | 35 | 2 | 2 | 1.82 | 1.77 | 0.05 | -0.25 | 0.34 | 885.5 | 0.920 | 0.946 | 0.01 | Q27 |
| Fig. 5C, Row 5 | 50 | 35 | 2 | 2 | 1.82 | 1.63 | 0.19 | -0.10 | 0.50 | 956 | 0.420 | 0.773 | 0.09 | Q28 |
| Fig. 2J | 46 | 39 | 2 | 2 | 2.09 | 1.85 | 0.24 | -0.19 | 0.64 | 1015.5 | 0.257 | 0.694 | 0.12 | Q29 |
| Fig. 5D, Row 2 | 16 | 16 | 2 | 1.5 | 1.94 | 1.63 | 0.31 | -0.13 | 0.81 | 160 | 0.179 | 0.538 | 0.24 | Q30 |
| Fig. 5D, Row 1 | 16 | 16 | 2 | 2 | 1.94 | 1.75 | 0.19 | -0.19 | 0.63 | 143.5 | 0.487 | 0.773 | 0.12 | Q31 |
| Fig. 5C, Row 6 | 39 | 35 | 3 | 2 | 2.64 | 2.06 | 0.58 | 0.05 | 1.11 | 870.5 | 0.036 | 0.194 | 0.24 | Q32 |
| Fig. 5C, Row 7 | 39 | 35 | 2 | 1 | 1.74 | 1.49 | 0.26 | -0.12 | 0.65 | 804 | 0.141 | 0.538 | 0.17 | Q33 |
| Fig. 2G | 40 | 35 | 2 | 1 | 1.83 | 1.51 | 0.31 | 0.05 | 0.57 | 887 | 0.025 | 0.194 | 0.26 | Q5 |
| Fig. 3B | 30 | 23 | 2 | 2 | 2.23 | 1.70 | 0.54 | 0.05 | 1.01 | 456 | 0.035 | 0.194 | 0.29 | Q6 |

**S2 Table. Comparison of survey responses between newly recruited (NEW) and previously enrolled (DSP-enrolled) participants**. Survey responses from (NEW) and those previously enrolled (DSP-enrolled) were compared using Mann-Whitney U tests. Analyses were restricted to survey items with sufficient responses across groups. Questions that were only administered to authors were excluded due to the limited number of author respondents (NEW, n = 7; DSP-enrolled, n = 4). For all other questions, responses from all participant roles (*Quality/Impact* reviewers, *Impact*-only reviewers, authors) were pooled within the NEW and DSP-enrolled groups and compared. Lower scores corresponded to more favorable responses. P-values were adjusted using the Benjamini–Hochberg procedure. Effect sizes (r) were calculated from the standardized Mann-Whitney statistic as r = Z/√N, where Z was derived using the normal approximation with continuity and tie corrections and N is the total number of observations.
