# Supplementary material for: Discovery Stack Pilot demonstrates the feasibility and outcomes of a scientist-designed peer-review model that separates quality and impact
Source: PLoS Biol. 2026 Sep 8;24(9):e3003986. doi: 10.1371/journal.pbio.3003986 (PMC13614658; doi:10.1371/journal.pbio.3003986)
Supplement: Supplementary file 2 [file pbio.3003986.s013.docx]

We thank all the reviewers for their helpful comments.

------------------------------------

REVIEWERS' COMMENTS:

Reviewer #1: 
[identifies himself as Ross Mounce]

Those who seek to critique the current academic publication system really ought to make sure that their work is robust, well designed, and well evidenced. In my opinion this manuscript falls very short in terms of scientific quality. 

The experimental design of this manuscript is obviously flawed for many different reasons including:
A) insufficient sample size 
B) underexplained highly subjective recruitment, making it prone to possible bias
C) duration: trying to analyse the experiment too early before all the results are known
D) lack of demographic transparency or stratification in the sample recruitment 

Many of these experimental design issues could have been pointed out and corrected before any data was collected (and effort wasted?) if the researchers behind this study had submitted their experimental design as a registered report for peer review. I am thus also unsurprised to note that the authors appear to make no mention to or reference of registered reports in their manuscript. The authors might want to read a review article on registered reports: Chambers, C.D., Tzavella, L. The past, present and future of Registered Reports. Nat Hum Behav 6, 29-42 (2022)

I shall try to explain the perceived experimental design flaws in more detail below:

A) **Sample size** 
The recruitment of merely 18 preprints, only 17 of which could be included in all analyses is bafflingly small and makes this research statistically underpowered assuming the effect sizes are small (e.g. if Cohen's d = 0.2). I can see that this is labelled as a "pilot" and thus can't be expected to be an industry-wide experiment but equally I'd ask what good is such a tiny and insufficiently described experiment? Did the authors do any kind of prospective power analysis before collecting data to see how many preprints they ought to recruit to get meaningful results?

The small overall sample size is further undermined by the fact that the sample was recruited in three different ways. On of the recruitment methods recruited just three preprints, so a sample size of just three for that subset. Does the recruitment method not have an impact? I think it does. What sample size for each of the recruitment methods would you need to prove me wrong?

What if I were to assert that two of the recruited preprints were highly atypical papers? This is why you need a larger N for the number of recruited preprints, to accommodate for any outliers that might be in the sample.

**Response:** We thank the reviewer for these thoughtful concerns regarding sample size, recruitment, and study design. We agree that larger studies will be essential to evaluate generalizability and to detect small effect sizes.

This study was designed as a **pilot** to assess the **feasibility** and **implementation** of the Discovery Stack model, rather than to provide a definitive, fully powered evaluation of its effects. Accordingly, a prospective power analysis was not undertaken because the study was not structured to test a single, predefined hypothesis with an expected effect size, but rather to evaluate feasibility, workflow, reviewer engagement, and participant perceptions across multiple dimensions. We edited the manuscript to clarify these goals, and to emphasize that the results should be interpreted in the context of a pilot study.

The modest sample size of this pilot study was primarily driven by practical constraints in recruitment. Recruitment of both authors and reviewers emerges as a major limitation. Participation rates among individuals with no prior familiarity with the study were low, and recruitment of both authors and reviewers was strongly dependent on network-based outreach, which limited the achievable sample size despite extensive recruitment efforts. As clarified in the response to point B below, recruitment methods were designed to enable sufficient participation to conduct the pilot, rather than to compare recruitment methods.

Despite these limitations, several key insights emerged from this pilot that we believe warrant publication and further investigation. Specifically, we found that reviewers were able to evaluate *Quality* independently from *Impact*, that *Impact* assessments exhibited greater variability than *Quality* assessments, and that successful recruitment depended strongly on targeted outreach and prior familiarity. Importantly, participants strongly endorsed core elements of the model, including standardized metrics, in-line annotation, peer-improvement mindset, and distinct *Quality* and *Impact* evaluations, which together enhanced the clarity and constructiveness of peer reviews.

To address concerns regarding variability and potential influence of individual manuscripts, we incorporated multiple complementary analyses, including evaluation of dispersion metrics, bootstrap resampling, and subsampling, which are less sensitive to unequal sample sizes and allow estimation of uncertainty.

We agree that registered reports represent a valuable mechanism for improving methodological rigor, and we thank the reviewer for highlighting this literature. We added discussion of registered reports to the manuscript and identify this as an approach worth considering for future, larger-scale evaluations of the Discovery Stack model. We note, however, that the present pilot was iterative by design, with several procedural elements refined during recruitment in response to participant feedback. While this flexibility is well suited to a feasibility study, it is less compatible with the pre-registered, hypothesis-driven structure of a registered report.

B) **Underexplained highly subjective recruitment**

Why were three different recruitment methods employed?
1) Editor emailed DSP-enrolled authors
2) Editor emailed BioRxiv authors
3) Selected from BioRxiv - authors did not enroll

In category 3 can we please have more detail on how and why three were "selected by the pilot's scientific advisory board and reviewed without author input". There were over 43,000 preprints posted to BioRxiv in 2024, why were the three selected arbitrarily chosen out of thousands of other possible choices?

**Response:** We thank the reviewer for noting that the description of recruitment methods in the original manuscript was not sufficiently detailed. This helpful comment prompted us to substantially revise the text to clarify both the recruitment strategy and its underlying rationale.

**Recruitment strategy and sources.** What was originally presented as three enrollment methods is more accurately described as a single recruitment approach applied to two participant populations. The recruitment approach is an email-based invitation to corresponding authors. The two different populations that we reached out to include:

1) Previously enrolled Discovery Stack participants, who were asked whether they and an eligible preprint that had not yet been accepted for journal publication.
2) Corresponding authors of recent bioRxiv preprints meeting predefined eligibility criteria (described below).

The third category described in the original manuscript as “bioRxiv manuscripts reviewed without author input” does not represent a separate recruitment method, but rather a subset of bioRxiv authors who did not respond to our invitation. Since reviewers with appropriately matched expertise had already been identified and agreed to participate, these manuscripts were included in the pilot, despite the lack of author participation. We also corrected the number of manuscripts reviewed without author input from three to two, as the authors of one preprint later engaged with the process and provided feedback.

**Manuscript Selection Criteria**

In all cases, manuscripts were identified using predefined criteria:

1) Posted to a preprint server such as bioRxiv
2) Submitted to a traditional journal for publication
3) In the fields of immunology or cancer biology

These criteria were applied prospectively to enable testing of the in-line commenting tool, comparison of the Discovery Stack model to traditional review, and leverage the subject matter expertise of the scientific advisory board and available reviewers.

I suggest a more rigorous experimental design would have been to use a random number generator to randomly select bioRxiv preprints for recruitment into this experiment. "Three additional bioRxiv manuscripts were selected by the scientific advisory board…" leaves this study open to accusations of cherry-picking and bias in how these three extra preprints were selected.

Another bias is in category 1 ("Editor emailed DSP-enrolled authors") this is textbook self-selection bias. These DSP-enrolled authors have already shown interest and engagement in the Discovery Stack Project and so these are included? Would it not be a stronger test of the discovery stack model to recruit authors/preprints who have not heard of or previously engaged with the discovery stack model? The inclusion of DSP-friendly or DSP-aware authors in the experiment invalidates some of the results such as on page 10: 

"Moreover, 84.9% of authors and reviewers agreed that separating these dimensions led to more constructive and insightful evaluations than traditional reviews" 

because of self-selection bias. You're sampling from people who like the idea and so it's unsurprising that these authors might agree with reviewers who have also agreed to test the discovery stack model. Homophily.

**Response:** We agree with the reviewer that differences in prior familiarity with the Discovery Stack model could influence perception-based outcomes and that this is an important consideration when interpreting the results and designing future studies.

While random selection of preprints would be ideal for assessing generalizability and minimizing potential selection effects, the primary goal of this pilot was to evaluate feasibility and implementation of the Discovery Stack model, including the practical challenges of recruiting authors and reviewers. In practice, achieving sufficient participation proved to be a major challenge.

An important finding of this pilot was that participation was strongly dependent on prior familiarity and network-based outreach. Only 8% of individuals unfamiliar with the study accepted reviewer invitations, compared with 68% of previously engaged participants. Similarly, among authors, 16 of the 18 enrolled manuscripts (89%) were submitted by authors who were either already familiar with the study or were recruited through follow-up communication from a colleague familiar with the project. These data suggest that, without leveraging existing networks, a purely randomized recruitment strategy would likely have resulted in very low participation and prevented completion of the pilot. This finding highlights a broader challenge for implementing new peer review platforms, namely that successful recruitment may depend heavily on prior familiarity and targeted outreach. Accordingly, the inclusion of previously engaged participants and the focus on specific scientific domains was not intended to bias the study population, but rather reflected practical constraints necessary to execute the study.

To directly assess the concern that prior familiarity may influence responses, we performed an additional analysis comparing survey responses from participants new to the study with those previously enrolled in the pilot. This analysis showed that, for most survey questions, responses were highly similar between new and previously enrolled participants, with comparable median scores and uniformly small effect sizes. Visualization of mean differences with bootstrap confidence intervals demonstrated that most comparisons were centered near zero with overlapping confidence intervals. After adjustment for multiple comparisons, only one question showed a statistically significant difference between new and previously enrolled participants. This question assessed willingness to submit a manuscript to a similar platform in the future and did not directly evaluate perceptions of the review model itself. Moreover, as discussed in the manuscript, willingness to submit manuscripts to a new platform was lower than willingness to use such a platform for review and curation, even among participants familiar with the model. This finding highlights a key challenge for implementing a new peer review platform, as decisions about manuscript submission remain strongly influenced by traditional journal impact factors as a primary metric of researcher productivity.

The results of this analysis, now presented in Figure S2 and Table S1, suggest that prior familiarity with the Discovery Stack model did not meaningfully influence participant responses. Additionally, we revised the manuscript to clarify the recruitment approach and to note the limitation that, because participation was voluntary, the study population may be more receptive to alternative peer review models than the broader scientific community.

C) **Duration**
There's a remarkable degree of impatience displayed in this manuscript: "only six of 18 manuscripts had been accepted at the time of analysis". Why not simply wait a few more months before publishing an analysis so that the ultimate journal venue of publication can be analysed for >90% of the recruited preprints? Why rush to publish so prematurely? I think knowing the final publication venue for >90% of the recruited preprints would be valuable data to include in any serious analysis of this model.

**Response**: We agree that final publication outcomes are informative data and valuable for understanding how manuscripts are ultimately positioned within the traditional journal system. However, in practice, the time required to reach final publication decisions is highly variable and often prolonged. At the time of resubmission, now, 12 of 18 manuscripts had been accepted for publication at a traditional journal, while the remaining manuscripts have been under review for extended periods, with an average of 18.4 months (range: 16-21 months). Even among accepted manuscripts, the time from submission to publication averaged 13.5 months (range: 6-33 months). Thus, waiting for greater than 90% publication completion could require a prolonged and uncertain delay, potentially exceeding one to two additional years.

Importantly, many of the central findings of this study are not dependent on the final publication venue. The primary objectives of this pilot were to evaluate feasibility, reviewer behavior, and the performance of the Discovery Stack model itself, including the ability to distinguish *Quality* from *Impact*, variability in *Impact* assessments, and participant perceptions of structured review. These outcomes are intrinsic to the review process and are independent of downstream editorial decisions made by journals.

Additionally, a comparison of Discovery Stack reviews to destination journals is inherently limited by version asymmetry. Most manuscripts evaluated in the Discovery Stack pilot were initial submissions, whereas published versions include author revisions. As a result, such comparisons involve different versions of each manuscript rather than offering a like-for-like comparison. This asymmetry would persist regardless of how long we waited.

Accordingly, we believe that the current manuscript provides a meaningful and timely assessment of the model’s feasibility and performance. We made revisions to clarify the status of manuscript outcomes and to acknowledge that analysis of final publication venues remains an important area for future work as additional outcomes become available.

D) ** lack of demographic transparency or stratification in the sample recruitment ** 
The 18 preprints that form the core of this work appear to have been anonymised with labels such as DSPM118 and DSPL178. Why the anonymisation? If these are preprints published online at BioRxiv then they are published and known. I don't see why they need to be anonymised. It decreases my trust in this research that I don't know the exact identity of the 18 preprints that were recruited.

As I think we all recognise, no two scientific preprints are the same - a preprint isn't a simple substitutable unit. 
What are the demographic variations of each of these preprints?
i.e. How many authors are named on each preprint?
How many pages are each preprint? 
How many different institutions are affiliated with each paper? E.g. (1-100+)
Which countries are the listed author institutions on each preprint?

You can't call the discovery stack model a success if it's only been piloted with US & UK based academics… There needs to be explicit sociopolitical stratification built into the experimental design of the testing.

**Response**: We thank the reviewer for raising this important point regarding transparency and characterization of the sampled manuscripts.

Manuscripts were anonymized for two reasons. First, when authors enrolled their manuscripts in the pilot, we did not obtain explicit permission to publicly link reviewer comments and assessments to specific manuscripts. Although the preprints themselves are publicly available, the Discovery Stack reviews include reviewer identities and detailed in-line comments that were not collected under conditions permitting public release. Second, several manuscripts remain under journal review, and we sought to avoid any potential influence on ongoing editorial processes. For these reasons, manuscripts were assigned internal identifiers (e.g., DSPM118) to allow consistent tracking while maintaining confidentiality.

With respect to demographic and structural variation across manuscripts (e.g., number of authors, institutional diversity, and geographic distribution), we agree that these are important considerations for evaluating generalizability. However, the primary objective of this pilot was to assess feasibility and implementation of the Discovery Stack model rather than to perform a stratified analysis across manuscript or author characteristics. As such, the study was not designed or powered to support meaningful comparisons across these dimensions.

We revised the manuscript to acknowledge this limitation and to highlight the importance of incorporating broader demographic and geographic diversity, as well as explicit stratification, in future larger-scale studies.

Aside from the issues with the experiment design, I also take issue with the near total absence of the importance of software in the discussion of Quality.

The authors state: "Quality refers to the rigor and reproducibility of the data supporting a study's conclusions"

Is that it? Really? Data only? 
What about the critical role of software? Software should really get a mention as being part and parcel of research quality. As has been said elsewhere "The future of research software is the future of research" [1] 7 out of 10 UK-based researchers agree with the statement that it's impossible to conduct research without software. [2]

[1] Chue Hong NP, Aragon S, Hettrick S, Jay C. The future of research software is the future of research. Patterns (N Y). 2025 Jul 11;6(7):101322. doi: 10.1016/j.patter.2025.101322. PMID: 40926964; PMCID: PMC12416084. 
[2] Simon Hettrick (2014) 

I specifically also examined the Quality Assessment Checklist given in supplementary figure 4 - there was no mention of software or code here either. Reading the Quality Assessment Checklist I inferred that it was devised and/or written by wet lab biologists: "identify experiments not repeated a standard of three times" being quite an obvious tell in this regard. If there is a desire to expand the discovery stack model to further disciplines then specific items like this will need to be revised...

I note that the word "code" features in Supplementary Figure 3 panel B and panel C and a throwaway mention on page 21 of the manuscript as something that was actually suggested by survey participants (well done to those participants that mentioned it!): "Other suggestions included easier access to raw data, code, and protocols; moderation of comments and vetting of users and reviewers; and an initial quality screen before review."

The discovery stack model absolutely must be amended to include data and software/code (with parity) within the consideration of research Quality. Examining the quality of research with no regard to its underlying software/code is madness. 
Poor code can completely invalidate research and thus code must be inspected for quality. See e.g. the famous Reinhart and Rogoff debacle in economics 
Herndon, Thomas & Ash, Michael & Pollin, Robert. (2013). Does High Public Debt Consistently Stifle Economic Growth? A Critique of Reinhart and Rogoff. Cambridge Journal of Economics. 38. 257-279. 10.1093/cje/bet075

**Response**: We agree with the reviewer that software and code are critical components of research *Quality* and should be considered alongside experimental design, data integrity, and analysis. We appreciate the reviewer highlighting this important point.

For completed reviews, evaluation of computational methods, including software and code, was implicitly included under “Assessment of experimental design and methodology” and “data integrity”. However, we acknowledge that this was not adequately stated, and that clearer articulation is warranted. We plan to edit the *Quality* Assessment Forms as well as the *Quality* Assessment Checklist to include review of software and code used, as well as availability of data and code.

We note that the Discovery Stack Pilot was intentionally limited to manuscripts in immunology and cancer biology to align with available reviewer expertise. As a result, many of the checklist items reflect common evaluation criteria in these fields, including expectations for experimental replication. We agree that this emphasis may give the impression of a “wet lab–centric” framework and that broader applicability will require expansion of these criteria.

Importantly, an important goal of this pilot was to identify aspects of the model that would require refinement before broader application. This reviewer's feedback on software and code coverage is a clear example of the kind of insight the pilot was designed to surface. We noted in the discussion that as the model is extended to additional disciplines, the Quality assessment items will need to be revised and tailored.

In response, we revised the manuscript to explicitly acknowledge the role of software and code in defining research *Quality* and to note the need for future iterations of the *Quality* Assessment Checklist to incorporate evaluation of computational methods, code *Quality*, and reproducibility. We agree that this will be essential for extending the Discovery Stack model across disciplines and for ensuring a comprehensive assessment of research *Quality*.

The unwise disregard of code is evident within these authors own manuscript. I note with displeasure that on page 33 of the manuscript being reviewed the authors write: 
"Bootstrap resampling was performed using custom Python scripts to assess the SD, range, and IQR of Quality and Impact scores"

Where are these Python scripts? This reviewer would very much like to see these Python scripts before being able to decide on the quality of this manuscript. It would be good if the authors could share the code behind their analyses at a trusted data repository such as Zenodo or Dryad.

* For the sake of reproducibility, what version of the GraphPad Prism software did the authors use and what version of NumPy?

**Response**: We thank the reviewer for emphasizing the importance of code and data availability, and we agree that access to code and underlying data is essential for reproducibility and transparency.

The source data for all figures have been deposited at Zenodo (<https://doi.org/10.5281/zenodo.20041225>), and the complete code, including procedures to evaluate SD, range, and IQR of *Quality* and *Impact* scores, has been made publicly available on Github (<https://github.com/mmcgargi/Discovery-Stack-Analysis>). The GitHub repository includes scripts sufficient to reproduce the reported analyses and figures.

During revision, we performed new analyses comparing survey responses between new and previously enrolled participants using R. For consistency and to consolidate all analyses into a single, fully documented, and shareable workflow, we also re-ran the previous comparisons of variance between *Quality* and *Impact* reviews in R. In the revised analysis, we implemented a subsampling approach, rather than bootstrap resampling, to better account for unequal numbers of *Quality* and *Impact* reviews withing manuscripts. For each manuscript, *Impact* scores were randomly subsampled to match the number of *Quality* scores, and variability metrics were recalculated across iterations. Importantly, the conclusions remain unchanged, with both analyses demonstrating greater variability in *Impact* scores compared to *Quality* scores. Because the analyses are now fully implemented in R, Python and NumPy are no longer part of the analysis workflow.

The methods section has been revised to include the following Data, Code, and Software Availability statement:

**Data, Code, and Software Availability**

Statistical analyses were performed in GraphPad Prism (version 11.0.1) or R (version 4.5.1 [2025-06-13]) using RStudio (version 2026.04.0+526). Surveys and assessment forms were administered using the Formaloo platform (https://www.formaloo.com). Figures were generated in Prism or R and assembled in PowerPoint. Source data for all figures are available at <https://doi.org/10.5281/zenodo.20041225> and the code for analyses is available at <https://github.com/mmcgargi/Discovery-Stack-Analysis>.

* Where is there a copy of the exact survey instrument deposited, detailing all the questions and possible answers that were distributed to the 93 individuals (page 33). This does not seem to be contained within any of the supplementary figures.

**Response**: We thank the reviewer for highlighting the importance of providing the survey instruments. The full survey instruments distributed to Authors, *Impact* reviewers, and *Quality/Impact* reviewers, including all questions, response scales, and answer options, are now available alongside the source data at Zenodo (https://doi.org/10.5281/zenodo.20041225) for permanent archival access.

* Curiously the author feedback survey is said to have been completed by 11 of 18 authors (61%). Preprints in the fields of Immunology and Cancer Biology do not tend to be authored by just a single person. Surely the feedback survey should poll ALL the authors of each preprint in order to get the views of all the authors? I would thus expect the total number of authors to be something like more than 100 not just 18.

**Response**: We agree that most manuscripts in immunology and cancer biology involve large multi-author collaborations. However, the post-review surveys were intended to capture feedback from the authors directly engaged with the Discovery Stack review process. In most cases, this consisted of the author responsible for enrolling the manuscript in the Discovery Stack Pilot.

Co-authorship of a multi-author preprint does not necessarily imply engagement with the review process. Many co-authors contribute to specific experiments or sections of a manuscript and may not have reviewed the Discovery Stack reviews in detail. We did not systematically assess which co-authors had read or discussed the reviews. Accordingly, surveys were distributed only to authors who enrolled the manuscript in the pilot to ensure that responses reflected direct experience with the platform and review process.

Limiting responses to a single author representative per manuscript also avoided disproportionate weighting of manuscripts with large author lists and prevented overlapping assessments of the same review experience, which would have inflated apparent sample size without adding independent observations. We clarified this approach in the revised Methods section. We acknowledge that capturing the perspectives of broader author teams is an interesting question for future, larger-scale studies, particularly to assess whether attitudes toward alternative review models vary across author roles within a manuscript.

* I note the manuscript only cites 19 other sources. Considering the topic I find it a little bit insufficient. More comparison and acknowledgement needs to be made to both registered reports and the publish, review, curate (PRC) model as relevant alternatives (that share important similarities) to the discovery stack model. Aczel et al (2021) should be cited to highlight the financial and time cost of current peer review systems.

Aczel, B., Szaszi, B. & Holcombe, A.O. A billion-dollar donation: estimating the cost of researchers' time spent on peer review. Res Integr Peer Rev 6, 14 (2021).

**Response**: We thank the reviewer for highlighting the Aczel reference and agree that a broader discussion of related peer-review innovations would improve the manuscript. Accordingly, the revised manuscript includes an expanded discussion describing how the Discovery Stack model compliments and extends several existing approaches, including soundness-only review, publish-curate-review, open review, and Registered Reports. In each case, we discuss the shared goals and important distinctions between these approaches and the Discovery Stack model. We have also incorporated the Aczel et al. (2021) reference.

* Lack of discussion of the relevance or not of Journal Impact Factor™ numbers created and sold by Clarivate. The authors describe Journal Impact Factor numbers as merely "flawed". I think this is somewhat of an understatement.

See e.g. 
Curry, S. (2012, August 13). Sick of Impact Factors. Reciprocal Space. [https://urldefense.com/v3/__https://doi.org/10.59350/xqrv5-7bv94__;!!LQC6Cpwp!odjs6dQU-jXz5pDJZOD0HQ8V4N828pl_5VCRL-gv6HENIMkjFHRePyhixqo0_VjMXgLjiufshCX5v_tdJuS-3ak$](https://urldefense.com/v3/__https:/doi.org/10.59350/xqrv5-7bv94__;!!LQC6Cpwp!odjs6dQU-jXz5pDJZOD0HQ8V4N828pl_5VCRL-gv6HENIMkjFHRePyhixqo0_VjMXgLjiufshCX5v_tdJuS-3ak$) <[https://urldefense.com/v3/__https://doi.org/10.59350/xqrv5-7bv94__;!!LQC6Cpwp!odjs6dQU-jXz5pDJZOD0HQ8V4N828pl_5VCRL-gv6HENIMkjFHRePyhixqo0_VjMXgLjiufshCX5v_tdJuS-3ak$](https://urldefense.com/v3/__https:/doi.org/10.59350/xqrv5-7bv94__;!!LQC6Cpwp!odjs6dQU-jXz5pDJZOD0HQ8V4N828pl_5VCRL-gv6HENIMkjFHRePyhixqo0_VjMXgLjiufshCX5v_tdJuS-3ak$)> 
Brembs B, Button K, Munafò M. Deep impact: unintended consequences of journal rank. Front Hum Neurosci. 2013 Jun 24;7:291. doi: 10.3389/fnhum.2013.00291. PMID: 23805088; PMCID: PMC3690355.
Dimitrov JD, Kaveri SV, Bayry J. Metrics: journal's impact factor skewed by a single paper. Nature. 2010 Jul 8;466(7303):179. doi: 10.1038/466179b. PMID: 20613817. 

**Response**: We agree with the reviewer that “flawed” significantly understates the limitations of Journal Impact Factors and warranted additional discussion. We have substantially revised both the Introduction and Discussion to provide a more comprehensive overview of the conceptual and statistical limitations of impact factors and their widespread use as proxies for the value and impact of individual manuscripts and researchers. The revised manuscript now incorporates the suggested references and discusses issues including highly skewed citation distributions, the influence of individual highly cited papers, and the broader consequences of relying on journal-level metrics for research assessment. We also discuss how these limitations motivate the development of manuscript-level Quality and Impact assessments that are independent of journal branding.

* The lack of significant correlation of (submitted) Journal Impact Factor™ numbers with the Quality assessment is a fascinating result and should be discussed with reference to Brembs work:

Brembs B. Prestigious Science Journals Struggle to Reach Even Average Reliability. Front Hum Neurosci. 2018 Feb 20;12:37. doi: 10.3389/fnhum.2018.00037. Erratum in: Front Hum Neurosci. 2018 Oct 09;12:376. doi: 10.3389/fnhum.2018.00376. PMID: 29515380; PMCID: PMC5826185. 

I am delighted that the authors get a result yet again confirming that the research quality of individual papers has no significant correlation with (submitted) Journal Impact Factor (Figure 2E). That is a key finding that more scientists and policymakers should take on board. Although the low sample size again is a bit unfortunate… Will this hold up with a larger sample size?

**Response**: We thank the reviewer for highlighting this finding and for drawing our attention to the relevant work by Brembs. We share the reviewer's enthusiasm for this finding and agree it deserves more prominent discussion. In response, we edited the results and expanded the Discussion to place this result in the context of previous studies suggesting that journal prestige is a poor proxy for the methodological rigor and reliability of individual studies, including the work by Brembs (2018). We also emphasize that, although our pilot study was limited by sample size, the findings are consistent with the broader literature questioning the use of journal-level metrics to evaluate individual manuscripts. We revised the Discussion to frame this result as preliminary evidence supporting manuscript-level assessment of scientific Quality independent of journal branding, while acknowledging that validation in larger and more diverse cohorts will be an important objective of future studies.

Reviewer #2:
[identifies himself as Stephen Curry]

This is a very interesting manuscript reporting the results of a pilot study of a peer review model that separates the assessment of quality and impact and enables the used of in-line reviewer comments. Although the scale of the pilot is relatively small (18 manuscripts assessed; 162 quality and/or impact reviews obtained) and focused on just two biomedical subfields (cancer biology and immunology), it has been performed and reported very thoroughly - albeit a little too thoroughly in some repects, given the small numbers in the surveys that make up a large part of the analysis. That said, overall, it makes for a valuable contribution to the literature on peer review which, event at this late stage, remains something of a black box.

**Response**: We thank the reviewer for this thoughtful and constructive assessment of our manuscript and for recognizing the value of this pilot study. We appreciate the reviewer's recognition that, despite the limited scale of the pilot, the study has been carefully performed and reported. We also agree that several sections of the manuscript would benefit from clearer emphasis on the study’s central findings and more concise presentation of preliminary survey-based observations. We substantially revised the manuscript to address each of the concerns as detailed below.

There are a number of weaknesses in the study that I think should be addressed before publication. 

The authors do a good job of showing that reviewers are able to meaningfully distinguish quality from impact (p8-9), but I wanted to see a clearer analysis of the claimed support for standardized metrics (p10). Although the methods for arriving at quantitative scores for quality and impact are explained clearly in the Methods section (i.e. using separate questionnaires with Likert-Scale questions), I think a brief outline of the assessment process should be presented alongside the results.

**Response**: We agree that the assessment process and generation of composite *Quality* and *Impact* scores should be more clearly described alongside the results. To address this, we added a concise overview of the scoring framework at the start of the section titled “**Reviewers Successfully Distinguished *Quality* from *Impact”.*** The first paragraph of this section describes the structured assessment forms completed by reviewers, including the criterion categories, the Likert-scale and multiple-choice question formats, and the weighting and scoring approach used to generate composite scores for quantitative comparison across reviewers and manuscripts.

It strikes me that the reported support for standardized metrics could well also arise from the fact that the questionnaires provide clearly defined criteria for the quality and impact assessments and that it is this clarity of criteria (still very rare in normal practice) that appealed to authors, alongside the scoring mechanism for arriving at the metrics.

**Response**: We agree with this important observation that participant support for standardized metrics likely reflects not only the generation of quantitative scores, but also endorsement of the structured assessment framework itself. To address this point, we added text to the Results and Discussion noting that such explicit guidance is uncommon in traditional peer review and may have contributed to perceptions of improved clarity, consistency, and transparency.

In fact, I think the use of defined criteria is such an important feature of the review protocol at the heart of the study that
Supplementary Figures 6 and 7 should be promoted to the main body of the manuscript, rather than being relegated to the supplementary information, where they will more easily be missed.

**Response**: We agree that the *Quality* and *Impact* assessment forms are central components of the Discovery Stack framework and should be more prominently displayed. Accordingly, the assessment forms previously included as Supplementary Figures 6 and 7 have now been moved to the main body of the manuscript (Figures 2 and 3).

Given the very small number of authors surveyed in the study, I think the manuscript authors need to be much more circumspect about concluding that "Identify disclosure enhances perceived transparency" (p11-12). They also place emphasis on the observation that 4/10 authors "stated that identified reviews were more constructive than anonymous reviews", seemingly overlooking their finding that 6/10 authors found there was no difference or that reviewers were less constructive. I think the finding here is that the results are inconclusive! In any case it should be emphasised that findings based on such small numbers must be regarded as extremely preliminary.

**Response**: We agree with the reviewer that conclusions regarding perceived transparency and constructiveness should be interpreted cautiously given the limited number of author survey responses and manuscripts.

In response, we revised the section heading and substantially edited the text throughout this section to highlight the small sample size and clearly emphasize the preliminary nature of the survey observations. Specifically, we modified the discussion of authors’ perception of constructiveness to acknowledge that the small number of author responses were mixed and warrant further study in larger cohorts. We also revised the concluding summary to clarify that the effects of reviewer identification on perceived transparency and engagement remain inconclusive within this pilot cohort and require further investigation in larger studies.

More broadly, in response to this and related comments, we also revised the Discussion to consistently frame the survey-based observations as preliminary and to emphasize the need for larger cohorts to evaluate any of these effects with statistical confidence.

In many places the survey results are quoted to 3 significant figures. Given the small number of participants involved (<100), I think that a maximum of 2 significant figures would be more appropriate. This would also have the advantage of making the manuscript more readable.

**Response:** We agree with the reviewer that reporting survey results to three significant figures implies a level of precision not supported by the small sample sizes. In response, survey-based percentages and derived statistics throughout the manuscript were revised to use two significant figures, where appropriate. In cases with particularly small sample sizes (n ≤ 20), we additionally reported the raw counts (e.g., "four of 10 authors") to make the limited precision explicit. We agree this change also improves readability.

On p13-14 the section titled "Discovery Stack Platform Delivers a Better Experience than Traditional Review" discusses authors' and reviewers' perceptions of their new platform. When discussing the authors' responses, I think it would be worth considering the fact that the review process did not produce an accept/reject decision and discussing whether that might colour author perceptions.

**Response:** We agree with the reviewer that the absence of an editorial accept or reject decision may have influenced author perceptions of the Discovery Stack experience. To address this point, we added text to the manuscript acknowledging that, unlike traditional peer review, the Discovery Stack Pilot did not involve editorial publication decisions, which may have contributed to more favorable author perceptions of the platform experience.

At the top of p14 it is stated that "Most participants (75%) agreed that the platform fostered this mindset more effectively than traditional reviews". However, I would question whether the platform is solely responsible for this perception. In the Methods it is revealed that the briefing given to reviewers "emphasized the value of a peer-improvement mindset". At the very least, this instruction would appear to be a confounder of the analysis. The authors should remind the reader in presenting the results that this instruction was given and comment on its likely impact. 

**Response:** The reviewer raises an important point regarding the distinction between the technical platform itself and the broader Discovery Stack review framework. Throughout the manuscript, and in the survey, we used the term “platform” to refer to the entirer Discovery Stack model, including reviewer onboarding, structured assessment forms, in-line commenting, and explicit guidance emphasizing a peer-improvement mindset, rather than the software interface alone. We agree this terminology was not clear in the original manuscript. In the revised manuscript, we replaced “platform” with “model” where we refer to the broader Discovery Stack approach and use platform to refer to the technical structure. We also explicitly note that reviewer onboarding and guidance promoting constructive, peer-improvement–oriented feedback were intentional components of the model design and likely contributed to participant perceptions of the review experience.

On p18-19 there is a section titled "Expedited Review Is Achievable with Reviewer Accountability and Backup Plans". The purpose of this section is not clear to me. The authors report review times for quality and impact assessments, but then claim that their pilot cannot be compared directly to traditional review. They then proceed to discuss the impact of single late reviews and the need for contingency plans. In the absence of an experiment designed to directly compare traditional review with the Discovery Stack model, I don't see that this section adds any great value to the manuscript.

**Response:** We thank the reviewer for this comment and appreciate the opportunity to clarify the rationale for this section. One of the major goals of the Discovery Stack Pilot was to evaluate whether the review workflow could reduce review duration relative to traditional peer review, which remains a major limitation of the current publication system. Although the Discovery Stack process included two distinct review phases and therefore was not directly comparable in total duration to traditional review, the *Quality* review phase was designed to approximate the initial stage of journal review and was therefore compared to author-reported journal review timelines.

Importantly, the purpose of this section was not simply to report review times, but to identify operational bottlenecks that limited acceleration of the review process despite workflow simplifications intended to reduce reviewer burden. Specifically, the pilot revealed that delays were typically driven by isolated late reviewers rather than widespread reviewer noncompliance, leading to the conclusion that reviewer accountability and contingency planning are likely critical for achieving substantially faster peer review.

In response to the reviewer’s comment, we revised the section to more clearly state that the rationale for these analyses was to test whether the Discovery Stack model could accelerate the peer review process. Further, we clarified why identifying the causes of review delays is important for future implementation of the model.

The final two sections of the results (p20-21) probe the surveys for their interest in new publishing models and their views on desirable features. Again, given the small sample size, I don't think too much can be made of these results. Moreover, the answers to questions about using 'scientist-developed metrics to assess quality and impact' must be difficult to interpret if the form of the metrics is not discussed.

**Response:** We thank the reviewer for this thoughtful comment and agree that the survey-based findings should be interpreted cautiously given the modest cohort size. In response, we substantially shortened and consolidated these sections into a single, more focused section that presents the survey findings as supportive contextual observations rather than major conclusions.

We also revised the text to clarify that the “scientist-developed metrics” refer to structured, predefined evaluation criteria used to assess rigor (*Quality*) and significance (*Impact*), rather than unspecified numerical metrics alone. In addition, we explicitly added language acknowledging the limitations of the cohort size and the potential for selection bias among participants receptive to alternative peer review models.

We are told also in the last section (p21) that 71.8% of respondents would prioritise "evolving impact metrics based on ongoing community input". Maybe so - and I agree that impact is very difficult to assess a priori - but how would such "community input" be gathered? In the absence of any realistic prospect for a mechanism, such aspirations come across as somewhat naïve. I think here and in the discussion the authors should discuss the practical obstacles to "dynamic impact metrics".

**Response:** We thank the reviewer for raising this important question. We agree that the mention of "evolving *Impact* metrics" in the original manuscript did not adequately describe how such metrics would be generated in practice. We revised the Discussion to specify a concrete mechanism.

The dynamic *Impact* metric envisioned for future implementations would not require active solicitation of community opinion or new infrastructure built from scratch. Rather, it would integrate three existing or readily available signals: 1) reviewer *Impact* scores generated by the Discovery Stack model, capturing expert assessment of significance at the time of review; 2) citation counts drawn from established bibliometric databases to capture formal scientific uptake; and 3) altmetric indicators, including article downloads, reader saves on reference management platforms, social media engagement, reshares, press coverage, policy citations, and mentions in clinical guidelines, capturing readership, sharing, and broader societal influence. Each component is drawn from existing infrastructure, so the proposed metric is grounded in mechanisms already in operation rather than requiring novel community-curation tools.

The Discussion section also raises the possibility that in time "a threshold for high-Quality papers may emerge" from the use of standardized metrics. Although the authors are careful to acknowledge the risks associated with metrics, they don't outline a process for re-scoring quality on their platform. As presented, it produces a quality score for the submitted, not the revised manuscript. Re-scoring would add a further burden on reviewers, as would any attempt to keep track of impact months or years after publication. In any case, does this add substantial value above and beyond the practice of open review (in which reviews are published)? This should be discussed.

**Response:** We agree that the original Discussion did not adequately specify how *Quality* scores would evolve across manuscript revisions. In future implementations of the Discovery Stack model, revised manuscripts would undergo a second round of review by some or all of the original *Quality* reviewers, analogous to the multi-round structure of traditional peer review, with a new *Quality* score assigned to the revised version. The score displayed on the platform would reflect this updated assessment of the most recent manuscript version. Because this mirrors the structure of conventional peer review, the additional reviewer burden would not exceed that of the current system. We have clarified this in the revised Discussion.

We agree that open peer review, in which reviewer comments are published, shares important goals with the Discovery Stack model and has been a valuable advance in peer review practice. On reflection, we did not sufficiently emphasize what we consider to be one of Discovery Stack’s most significant advantage over open review, which is that the standardized *Quality* and *Impact* scores would function as decision aids that help readers identify rigorous and significant work amid a rapidly expanding body of literature. Open review publishes reviewer commentary, but a reader must still read through the full review text of each paper to assess its rigor and significance. By contrast, standardized, comparable metrics allow readers to filter, search, and prioritize at scale, which is increasingly important as the number of journals and published articles continues to grow. In addition to this core advantage, the Discovery Stack model provides explicit separation of *Quality* from *Impact*, defined assessment criteria, and integration of post-publication *Impact* tracking. We revised the Discussion to make these distinctions more explicit.

The discussion of the "broad dissatisfaction with current publishing norms" seems to me to add nothing new to our understanding of present woes and could be truncated. Happy to be corrected if I have missed something. 

**Response:** We agree with the reviewer that the discussion of dissatisfaction with current publishing norms largely restated well-documented concerns and did not add substantially to existing understanding. We truncated this discussion considerably. The revised paragraph now notes only briefly our cohort's dissatisfaction with the peer review system, and instead focuses on the more novel finding: the specific features participants identified as most likely to drive adoption of an alternative model, which align closely with the Discovery Stack model.

Overall, I have the impression that the numerous and detailed analyses presented in this paper end up muddying or masking what I think are its most important messages. These are the demonstrable value of a review protocol that separates quality from impact, AND assesses these using multiple, clearly defined criteria scored in a standardised way. The second important innovation is the utility of introducing in-line reviewer comments. I think some of the weaker or less important results sections could be truncated (or relegated to the supplementary information) and this would allow the central findings to come through more strongly.

**Response:** We appreciate this insightful feedback and agree that the manuscript is strongest when focused on the separation of *Quality* from *Impact*, the use of structured standardized evaluation criteria, and the incorporation of in-line reviewer comments.

In response, we shortened and consolidated several of the more exploratory survey-based sections in the Results and Discussion, reduced interpretive emphasis on these findings, and reframed them as supportive observations rather than primary outcomes. We also moved the criteria used to assess *Quality* and *Impact* from supplementary figures to main text Figures 2 and 3, so that the structured, criterion-based assessment framework is prominently visible rather than relegated to supplementary information. Additionally, we added a new paragraph in the results describing the assessment process and development of composite scores. Together, these revisions sharpen the manuscript’s focus on the core conceptual and operational advances demonstrated by the pilot study.

It would also help to have a stronger focus on the practicalities, given the well-known pressures on the peer review system. A protocol that needs 2-3 quality reviewers and 5-6 impact reviewers doesn't seem likely to take off. Would it be more realistic to have fewer reviewers, who both score quality and impact (using the different criteria) and down-weight the impact score given the greater uncertainty associated with that assessment? A sharper consideration of the practicalities would in my view enhance the quality of the present manuscript and augment the probability that it will have a significant impact!

**Response:** We thank the reviewer for these practical considerations, which are central to whether a model like this could realistically be adopted.

We note that the Discovery Stack model already incorporates part of the reviewer's suggestion. *Quality* reviewers assessed both *Quality* and *Impact*, completing a *Quality* assessment followed by a separate *Impact* assessment of the same manuscript using the distinct criteria for each dimension. The additional *Impact*-only reviewers supplemented the *Impact* assessments. We recognize that the original manuscript did not make this dual role of *Quality* reviewers sufficiently clear, and we revised the Results section accordingly.

The rationale for recruiting additional *Impact*-only reviewers was that *Impact* assessments are inherently more subjective and variable than *Quality* assessments, which was supported by our data. Because the goal of the *Impact* assessment is to capture the range of perspectives on a manuscript's significance, a larger number of *Impact* assessments provides a more representative distribution of those perspectives. In this framing, the variability of *Impact* is not noise to be minimized but information to be characterized, which argues for more, rather than fewer, *Impact* assessments.

It is also important to note that the *Impact*-only review is a substantially lighter task than the *Quality* review. *Impact*-only reviewers receive the *Quality* reviewers' assessments of rigor, which enables them to focus solely on evaluating significance rather than re-assessing the underlying science. Consistent with this, *Impact*-only reviews required an average of only 1.7 hours to complete. The additional *Impact* reviewers therefore add less to the total reviewer burden than their number alone would suggest.

We fully agree that reviewer recruitment and burden are critical practical constraints, and we expanded the Discussion to address these considerations.

Reviewer #3:
[identifies himself as Ludo Waltman]

My review report is available online: <https://urldefense.com/v3/__https://prereview.org/reviews/18915960__;!!LQC6Cpwp!odjs6dQU-jXz5pDJZOD0HQ8V4N828pl_5VCRL-gv6HENIMkjFHRePyhixqo0_VjMXgLjiufshCX5v_tdqJM4kn0$> <[https://urldefense.com/v3/__https://prereview.org/reviews/18915960__;!!LQC6Cpwp!odjs6dQU-jXz5pDJZOD0HQ8V4N828pl_5VCRL-gv6HENIMkjFHRePyhixqo0_VjMXgLjiufshCX5v_tdqJM4kn0$](https://urldefense.com/v3/__https:/prereview.org/reviews/18915960__;!!LQC6Cpwp!odjs6dQU-jXz5pDJZOD0HQ8V4N828pl_5VCRL-gv6HENIMkjFHRePyhixqo0_VjMXgLjiufshCX5v_tdqJM4kn0$)> .

Ludo Waltman
March 8, 2026

FLAT TEXT VERSION OF THAT REVIEW:
This paper presents the outcomes of a pilot study of a new approach to peer review of research articles. In this new approach, the quality and impact of an article are evaluated separately. I found the paper highly interesting to read. As pointed out by the authors, established approaches to peer review are facing major challenges. There is a strong need for studying alternative approaches to peer review.

My comments on the paper are fairly minor.

My most important comment is that the idea of separating the evaluation of quality and impact is not new. The authors should acknowledge existing approaches based on this idea. Let me highlight two approaches.

First, an increasing number of journals are using a so-called soundness-only approach to peer review. These journals ask reviewers to evaluate the soundness (i.e., quality) of an article and to refrain from evaluating the impact of an article. Soundness-only peer review was pioneered by PLOS One. Nowadays it is also used by various other journals, such as Scientific Reports. There is also literature studying soundness only peer review. See [https://urldefense.com/v3/__https://doi.org/10.1108/JD-06-2017-0092__;!!LQC6Cpwp!odjs6dQU-jXz5pDJZOD0HQ8V4N828pl_5VCRL-gv6HENIMkjFHRePyhixqo0_VjMXgLjiufshCX5v_td3WYCEAc$](https://urldefense.com/v3/__https:/doi.org/10.1108/JD-06-2017-0092__;!!LQC6Cpwp!odjs6dQU-jXz5pDJZOD0HQ8V4N828pl_5VCRL-gv6HENIMkjFHRePyhixqo0_VjMXgLjiufshCX5v_td3WYCEAc$) <[https://urldefense.com/v3/__https://doi.org/10.1108/JD-06-2017-0092__;!!LQC6Cpwp!odjs6dQU-jXz5pDJZOD0HQ8V4N828pl_5VCRL-gv6HENIMkjFHRePyhixqo0_VjMXgLjiufshCX5v_td3WYCEAc$](https://urldefense.com/v3/__https:/doi.org/10.1108/JD-06-2017-0092__;!!LQC6Cpwp!odjs6dQU-jXz5pDJZOD0HQ8V4N828pl_5VCRL-gv6HENIMkjFHRePyhixqo0_VjMXgLjiufshCX5v_td3WYCEAc$)> .

Second, the journal eLife uses a publish-review-curate model in which an explicit distinction is made between the strength of evidence (i.e., quality) and the significance of the findings (i.e., impact). Strength of evidence and significance of the findings are each assessed on a five- or six-point scale. There seem to be close connections between the eLife model and the Discovery Stack model proposed by the authors. The authors should discuss these connections.

**Response:** We thank the reviewer for this important point and fully agree that the conceptual separation of scientific rigor from perceived significance has been explored through several recent innovations in scholarly publishing. In response, we substantially revised the Discussion to better position the Discovery Stack model within this evolving landscape. Specifically, we now discuss soundness-only peer review, pioneered by PLOS ONE and subsequently adopted by other journals, and cite the suggested literature describing both its goals and practical implementation. We also discuss the publish-review-curate model implemented by eLife, including its distinction between strength of evidence and significance of findings.

The revised Discussion emphasizes that the Discovery Stack model builds upon these prior innovations rather than introducing the concept of separating rigor from impact de novo. We also highlight important distinctions between these approaches and the Discovery Stack framework. In particular, Discovery Stack explicitly generates independent, standardized Quality and Impact metrics, incorporates in-line annotation, combines transparent review with manuscript-level scoring, and provides a framework for dynamic post-publication evaluation of scientific influence. We believe these revisions more clearly acknowledge prior work while clarifying the unique contributions and intended role of the Discovery Stack model within the broader ecosystem of peer review innovations.

A minor comment relates to the way in which the authors use the word ‘published’. The authors for instance state: “At the time of analysis, only six of 18 manuscripts had been published. The mean from submission to publication for the published manuscripts was 231 days.” This is confusing. Articles can be published (i.e., be made publicly available) not only in journals but also on other platforms, such as preprint servers. In this case, all 18 manuscripts have already been published on a preprint server. Therefore, instead of “only six of 18 manuscripts had been published”, the authors should say “only six of 18 manuscripts had been published in a journal”. Likewise, “from submission to publication” should be “from submission to publication in a journal”.

**Response**: We agree that all 18 manuscripts in the pilot had already been published as preprints at the time of recruitment. Therefore, we revised the manuscript throughout to clarify this by using "published in a journal" where journal publication is intended.

Finally, I was unable to find the data underlying the analyses presented in the paper. In the interest of transparency and reproducibility, publishing the data is crucial.

**Response**: We thank the reviewer for emphasizing the importance of code and data availability, and as noted above, we agree that access to code and underlying data is essential for reproducibility and transparency. Response: We thank the reviewer for highlighting this important distinction.

The methods section has been revised to include the following Data, Code, and Software Availability statement:

**Data, Code, and Software Availability**

Statistical analyses were performed in GraphPad Prism (version 11.0.1) or R (version 4.5.1 [2025-06-13]) using RStudio (version 2026.04.0+526). Surveys and assessment forms were administered using the Formaloo platform (https://www.formaloo.com). Figures were generated in Prism or R and assembled in PowerPoint. Source data for all figures are available at <https://doi.org/10.5281/zenodo.20041225> and the code for analyses is available at <https://github.com/mmcgargi/Discovery-Stack-Analysis>.
