# Supplementary material for: Discovery Stack Pilot demonstrates the feasibility and outcomes of a scientist-designed peer-review model that separates quality and impact
Source: PLoS Biol. 2026 Sep 8;24(9):e3003986. doi: 10.1371/journal.pbio.3003986 (PMC13614658; doi:10.1371/journal.pbio.3003986)
Supplement: Supplementary file 3 [file pbio.3003986.s014.docx]

**EDITOR’S COMMENTS:**

IMPORTANT - please attend to the following:

a) We need your Title to contain an active verb and to avoid a colon (these tend to result in title truncation by some aggregators). We suggest changing it to: "A pilot study reveals the feasibility and outcomes of Discovery Stack, a scientist-designed peer peview model that separates quality and impact"

**Response:** We revised the title to include an active verb and remove the colon. The new title is: “Discovery Stack Pilot Demonstrates the Feasibility and Outcomes of a Scientist-Designed Peer-Review Model That Separates Quality and Impact”

b) Please attend to the remaining comments from the reviewers.

**Response:** We addressed the remaining comments from all reviewers and revised the manuscript accordingly. Detailed responses to each comment are provided below. The modified text is tracked in Word.

c) Please confirm that this process, which involved human subjects in a trial peer review process, did not require ethical approval.

**Response:** We confirm that this study did not require ethical approval. The Discovery Stack Pilot evaluated a peer-review process involving voluntary participation by authors and reviewers and did not constitute human subjects research requiring institutional ethics review.

d) We note that you specify one author as receiving "specific" support for this project. Please also declare any non-specific support which was used for this project.

**Response:** There was no non-specific support for this project. All participants were volunteers (just as for typical peer review!). Only author McGargill derived salary from participation.

e) Please address my Data Policy requests below; specifically, we need you to supply the numerical values underlying Figs 1BC, 4ABCDEFGHIJ, 5ABCD, 6ABCD, 7ABC, 8ABCD, 9ABCDEFGHIJ, 10AB, S1CDEF, S2AB, S3ABCDEFGHIJKLM, S4ABCDEFGH, S5ABC, S7AB, either as a supplementary data file or as a permanent DOI’d deposition.

**Response:** All numerical values for the figures mentioned above are either in the supplemental tables or available at <https://doi.org/10.5281/zenodo.20041225>. Each figure now states where the data can be found.

f) Please cite the location of the data clearly in all relevant main and supplementary Figure legends, e.g. “The data underlying this Figure can be found in S1 Data” or “The data underlying this Figure can be found in https://urldefense.com/v3/__https://zenodo.org/records/XXXXXXXX__;!!LQC6Cpwp!q6kGxYJ1KExfPeLl8xJXWQuULJ_P9guNyYsnAW9EqqWO0eycFYIw6aucHQrsT8VxSHVZAKQfeeoR_HVJDWw0pUY$ ; ; also update your Data Availability Statement accordingly.

**Response:** Each figure now states where the data can be found, either in the supplemental tables or available at <https://doi.org/10.5281/zenodo.20041225>. The Data Availability Statement has been updated accordingly.

g) Please make any custom code available, either as a supplementary file or as part of your data deposition.

**Response:** The code for analyses is available at <https://github.com/mmcgargi/Discovery-Stack-Analysis> and is stated in the Data, Code, and Software Availability section.

h) Please include the URLs of your funders in the Financial Disclosure statement.

**Response:** Now done in the online portal

**REVIEWERS' COMMENTS:**

**Reviewer #1:
[identifies himself as Ross Mounce]**

Thank you for the extensive and thoughtful revision. I appreciate the care with which you have engaged with the reviewers' comments and the substantial effort invested in clarifying the study design, expanding the discussion, improving transparency, and making the underlying data and code available.

Having read both the original review reports and your detailed responses, I believe the manuscript has improved considerably. In particular, the additions regarding recruitment, the explicit framing of the work as a feasibility pilot, the new analyses addressing familiarity bias, the provision of code and survey instruments, and the expanded discussion of related initiatives (e.g., soundness-only review, publish-review-curate approaches, and Registered Reports) all strengthen the manuscript and address many of the concerns raised previously.

That said, I remain somewhat cautious about the strength of several conclusions and would encourage further moderation of the manuscript's framing in a few areas.

**1. Pilot framing and generalizability | Overclaiming**

The revised manuscript does a much better job of describing itself as a feasibility study, and I commend the authors for explicitly acknowledging recruitment challenges, selection effects, and the limited size of the cohort. However, I still feel that some passages occasionally drift from "evidence of feasibility" toward broader claims regarding the supposed superiority or future success of the model.

The central contribution of this paper is not that the Discovery Stack model has been proven superior to existing peer review systems. Rather, it is that the authors have demonstrated the feasibility of recruiting 18 independent co-authors of preprints into implementing a new review process that separates assessments of Quality and Impact, incorporates structured criteria, and uses in-line commenting. The manuscript is strongest when it stays focused on these achievements.

**Response:** We thank the reviewer for these thoughtful comments and for recognizing the substantial revisions made in response to the previous review. We appreciate the recommendation to further moderate several statements so that they remain fully consistent with the scope of a feasibility pilot study. We agree that the primary contribution of this work is demonstrating the feasibility of implementing the Discovery Stack review model and generating preliminary evidence regarding its operation and participant perceptions, rather than establishing superiority over existing peer review systems. In response, we further revised the manuscript to more clearly distinguish observations supported by the current pilot study and directions for future investigation.

For instance: 
*       the final sentence of the Discussion, the claim that the system "is ready for expansion and adoption" goes beyond what a pilot of 18 manuscripts can demonstrate. The study shows feasibility, not readiness for broad deployment.

**Response:** We thank the reviewer for this suggestion and agree that this statement extended beyond what can be concluded from a feasibility pilot. We revised the final sentence of the Discussion to emphasize that the Discovery Stack Pilot provides a foundation for future refinement and larger-scale evaluation, rather than indicating readiness for broad adoption.

*       Quote from the revised manuscript: "In future implementations of the platform, a threshold for high-Quality papers may emerge, above which manuscripts would be designated as rigorous and trustworthy."
o       This is an interesting hypothesis, but it implies a level of validation that has not yet occurred. The pilot demonstrates that reviewers can generate Quality scores; it does not yet establish that those scores are sufficiently calibrated, reproducible, or predictive to support a threshold denoting trustworthy science.

**Response:** We appreciate this comment. Our intention was not to suggest that the present pilot establishes or validates a threshold for scientific rigor, but rather to illustrate one potential long-term application of standardized *Quality* metrics. We agree that developing such thresholds would require substantially larger datasets and rigorous validation of score calibration, reproducibility, and predictive performance. We revised this section to clarify that this is a future goal that would build upon standardized *Quality* metrics, rather than a conclusion supported by the current pilot.

*       Quote from new text in the revised manuscript: "More examples would need to be produced but we consider it likely that a system like the one we tested would in the long run be at least as predictive of the long-term citation/value of a given piece of science as the journal impact factor."
o       This is tremendously speculative about the Discovery Stack model. I think it also manages to misrepresent the science of studies about Journal Impact Factor and its relation to the citedness of individual papers. Journal Impact Factor is a poor predictor of the citedness of individual papers within a journal. As shown by: Seglen, P.O. (1997). "Why the impact factor of journals should not be used for evaluating research." BMJ 314: 498-502. Zhang, L., Rousseau, R., & Sivertsen, G. (2017). "Science deserves to be judged by its contents, not by its wrapping." PLOS ONE 12:e0174205. Larivière, V. et al. (2016). "A simple proposal for the publication of journal citation distributions." and others...

**Response:** We appreciate this point and agree that the previous statement was overly speculative and extended beyond what could be concluded from the present pilot study. Accordingly, we removed this statement from the manuscript. We also moved the figure (**Fig 7S**) that accompanied this statement to the “**Reviewers Successfully Distinguished Quality from Impact**” Results section, where it serves to illustrate the observed relationship between reviewer-assessed Impact scores and JIF tiers, rather than supporting speculation regarding long-term citation patterns or future predictive performance. In the revised manuscript, this figure is now **Fig S2**.

**2. Recruitment bias remains an important limitation**

I appreciate the additional analyses comparing previously engaged participants and newly recruited participants. The finding that responses were broadly similar is useful and reassuring. Nevertheless, I do not think these analyses entirely eliminate concerns about participation bias.

The manuscript now documents that reviewer acceptance rates were dramatically higher among individuals already familiar with the project or contacted through existing networks. Indeed, the study reveals that personal outreach and prior engagement were the dominant determinants of participation.

To me, this is an interesting finding. It suggests that enthusiasm for alternative peer-review systems may not yet extend broadly across the wider research community and that adoption barriers remain substantial.

**Response:** We thank the reviewer for this thoughtful observation. We agree that the additional analyses comparing newly recruited and previously enrolled participants do not eliminate the possibility of participation bias. Rather, they provide insight into an important challenge associated with implementing alternative peer-review models. As described in the Results, reviewer recruitment was substantially more successful among individuals with prior familiarity with the Discovery Stack model or who received outreach through existing professional networks. In response to the reviewer's suggestion, we revised the Discussion to emphasize that these findings suggest that current enthusiasm for the model may not yet extend broadly across the wider research community, and that broader adoption will require strategies to engage scientists beyond early adopters and existing professional networks.

In the response to the reviewer comments, the authors disclose that only a single-author of each multi-author enrolled manuscript responded to the surveys. This masks variation in the role of each author in multi-author teams and how that maps to responses each author might give to the survey questions. What if the survey-responding author was a first-author, what if the survey-responding author was the PI on the grant and/or the last-author, what if the survey-responding author was a middle author amongst twenty other middle authors, who did the some of the pipetting work (minor involvement). It seems odd to treat all authors on a preprint as "the same", when particularly in terms of power, influence, and positionality towards change in scholarly communication, there are very important differences between researchers in different stages of their careers. This was not controlled for in the experimental design. Unfortunate.

**Response:** We thank the reviewer for raising this important point. We agree that treating all authors as equivalent could obscure meaningful differences in role, seniority, and involvement, which we were not able to address in this manuscript. In response, we revised the methods to clarify that the author survey was intentionally directed to a single representative author for each manuscript, specifically the senior author or first author, as these individuals are typically responsible for managing manuscript submission and responding to peer review. We also included here that among the 11 author respondents, nine were senior authors and principal investigators, while the remaining two author respondents were first authors (one postdoctoral fellow and one graduate student). Because of the small number of respondents, the pilot was not designed or sufficiently powered to evaluate differences in perceptions according to authorship role or career stage. We agree that this is an important consideration and added this limitation to the Discussion, noting that future studies should include larger numbers of authors and evaluate whether perspectives differ across authorship roles and career stages.

**3. dynamic Impact metrics**

The revised discussion provides a much clearer explanation of how dynamic Impact metrics might work in practice. I found this addition helpful.

That said, implementation remains speculative. Citation counts, altmetrics, expert evaluations, downloads, policy citations, and social engagement all have known limitations and biases. I would encourage the authors to make clear that the proposed framework remains aspirational and untested rather than implying that an accepted methodology already exists.

**Response:** We thank the reviewer for this helpful suggestion. We agree that the framework for dynamic Impact metrics is intended as a conceptual long-term vision rather than an established methodology. Our intention was to illustrate one possible direction for future development of the Discovery Stack model rather than imply that an accepted approach currently exists. Accordingly, we revised the Discussion to explicitly state that the implementation and validation of dynamic Impact metrics remain future objectives and that determining how best to integrate expert assessments, citation-based measures, and broader indicators of influence will require rigorous empirical evaluation.

\### Overall assessment

My overall view is considerably more positive than in the previous round.
While I remain unconvinced that this study provides strong evidence for the effectiveness of the Discovery Stack model at scale, I do believe it now provides useful evidence that 18 anonymised preprints have undergone a Discovery Stack paid-review process. The manuscript is more transparent-ish, more balanced, better contextualized within the broader literature, and substantially more reproducible than the version originally reviewed.

However, one should not overlook the impact of paying peer-reviewers on the assessment of the model feasibility ($30 for each Quality review, $20 for each Impact review). Was it only feasible because you were offering direct financial payment? Would this level of payment be feasible if scaled up? Who exactly would provide the funding to pay for the payments to peer-reviewers and would they be happy to pay these extra fees, at a time when the United States Office of Management and Budget is proposing to stop paying from federal funds for any publication-related costs?[1] I'm not at all surprised that many happy volunteers were found to pilot this model given that payment was offered. Ker-ching! People like to earn more money. The understandable desire from an individual to earn more money may perhaps mask actual enthusiasm for the scholarly benefit(s) or disbenefit(s) of the model?

[1] [https://urldefense.com/v3/__https://sparcopen.org/our-work/2026-proposed-2cfr200-updates-faqs/__;!!LQC6Cpwp!q6kGxYJ1KExfPeLl8xJXWQuULJ_P9guNyYsnAW9EqqWO0eycFYIw6aucHQrsT8VxSHVZAKQfeeoR_HVJVrUl76c$](https://urldefense.com/v3/__https:/sparcopen.org/our-work/2026-proposed-2cfr200-updates-faqs/__;!!LQC6Cpwp!q6kGxYJ1KExfPeLl8xJXWQuULJ_P9guNyYsnAW9EqqWO0eycFYIw6aucHQrsT8VxSHVZAKQfeeoR_HVJVrUl76c$)  &[https://urldefense.com/v3/__https://www.federalregister.gov/documents/2026/05/29/2026-10817/regulation-for-federal-financial-assistance__;!!LQC6Cpwp!q6kGxYJ1KExfPeLl8xJXWQuULJ_P9guNyYsnAW9EqqWO0eycFYIw6aucHQrsT8VxSHVZAKQfeeoR_HVJwihaY-0$](https://urldefense.com/v3/__https:/www.federalregister.gov/documents/2026/05/29/2026-10817/regulation-for-federal-financial-assistance__;!!LQC6Cpwp!q6kGxYJ1KExfPeLl8xJXWQuULJ_P9guNyYsnAW9EqqWO0eycFYIw6aucHQrsT8VxSHVZAKQfeeoR_HVJwihaY-0$)

**Response:** We thank the reviewer for raising this important point. Reviewer compensation was intentionally incorporated into the pilot because equitable compensation for peer-review labor is a central principle of the Discovery Stack model and was included as part of the model being evaluated, rather than solely as a recruitment strategy. We agree that compensation has the potential to influence participation. However, the present study was not designed to determine the relative contributions of compensation, prior familiarity with the project, professional outreach, or scientific interest to reviewer participation. In addition, 73% of reviewers (55 of 75) elected to donate their (modest, I’m sure we must all agree) compensation rather than retain it, suggesting that financial compensation was not the sole motivation for many participants. We revised the Discussion to clarify the rationale for including reviewer compensation in the pilot and to note that identifying sustainable funding mechanisms to support equitable reviewer compensation will be an important consideration for broader implementation of alternative peer-review models. We also edited the Methods to note that reviewers had the option to donate their compensation and report that 73% of reviewers (55 of 75) elected to do so.

Sidenotes:

A stylistic point. I would advise against lower-casing mentions of Clarivate's proprietary Journal Impact Factor ™ (JIF) numbers. Lower-casing gives the false impression that JIF numbers are somehow transparent, reproducible, or sensibly calculated. They are not. They are proprietary, negotiable, statistically-illiterate numbers which only Clarivate has the right to assign to journals as their official (Clarivate-awarded) Journal Impact Factor™ . References throughout to 'journal impact factor' or, even worse 'impact factor' [failing to mention the word journal, which neglects to explicitly inform that it is a journal-level number] may mislead readers into thinking that these are generic numbers created following some kind of rational and statistically-sound method - they are NOT!

**Response:** We thank the reviewer for this helpful suggestion. We agree that it is important to clearly distinguish the proprietary Journal Impact Factor (JIF) from more general measures of scientific impact. Accordingly, we revised the manuscript to define the Journal Impact Factor (JIF) at its first mention as a proprietary metric calculated by Clarivate and thereafter refer consistently to the JIF when discussing this journal-level metric. This revision also helps distinguish JIF from the manuscript-level Impact scores generated by the Discovery Stack model.

Prior examples of using in-line commenting for peer-review of preprints include one by myself in 2021[https://urldefense.com/v3/__https://greenelab.github.io/annorxiver_manuscript/__;!!LQC6Cpwp!q6kGxYJ1KExfPeLl8xJXWQuULJ_P9guNyYsnAW9EqqWO0eycFYIw6aucHQrsT8VxSHVZAKQfeeoR_HVJfMIe-I4$](https://urldefense.com/v3/__https:/greenelab.github.io/annorxiver_manuscript/__;!!LQC6Cpwp!q6kGxYJ1KExfPeLl8xJXWQuULJ_P9guNyYsnAW9EqqWO0eycFYIw6aucHQrsT8VxSHVZAKQfeeoR_HVJfMIe-I4$)  (see the Hypothesis logo in the top-right corner). This was journal-organized peer-review. The journal which organized it was PLOS Biology (!). The journal eventually published it here: [https://urldefense.com/v3/__https://journals.plos.org/plosbiology/article?id=10.1371*journal.pbio.3001470__;Lw!!LQC6Cpwp!q6kGxYJ1KExfPeLl8xJXWQuULJ_P9guNyYsnAW9EqqWO0eycFYIw6aucHQrsT8VxSHVZAKQfeeoR_HVJjIeU6Bo$](https://urldefense.com/v3/__https:/journals.plos.org/plosbiology/article?id=10.1371*journal.pbio.3001470__;Lw!!LQC6Cpwp!q6kGxYJ1KExfPeLl8xJXWQuULJ_P9guNyYsnAW9EqqWO0eycFYIw6aucHQrsT8VxSHVZAKQfeeoR_HVJjIeU6Bo$) 
These in-line [Hypothes.is](http://Hypothes.is) comments were incorporated into and extensively referenced in the submitted first-round peer review report[https://urldefense.com/v3/__https://journals.plos.org/plosbiology/article/peerReview?id=10.1371*journal.pbio.3001470__;Lw!!LQC6Cpwp!q6kGxYJ1KExfPeLl8xJXWQuULJ_P9guNyYsnAW9EqqWO0eycFYIw6aucHQrsT8VxSHVZAKQfeeoR_HVJ90W_1Tg$](https://urldefense.com/v3/__https:/journals.plos.org/plosbiology/article/peerReview?id=10.1371*journal.pbio.3001470__;Lw!!LQC6Cpwp!q6kGxYJ1KExfPeLl8xJXWQuULJ_P9guNyYsnAW9EqqWO0eycFYIw6aucHQrsT8VxSHVZAKQfeeoR_HVJ90W_1Tg$) 
I'm sure there are many other examples, either more systematically implemented at a journal, or at an earlier date than 2021, such as at the American Geophysical Union ([Hypothes.is](http://Hypothes.is) and the Geophysical Electronic Manuscript Submission [GEMS] in editorial review) and JMIR Publications ([Hypothes.is](http://Hypothes.is) and preprints). The point is that in-line commenting for peer-review of preprints during journal-organized peer review is not completely new to the Discovery Stack model. I just wanted to make that clear.

**Response:** We thank the reviewer for highlighting these important examples. We agree that the use of in-line annotation during journal-organized peer review predates the Discovery Stack model and did not intend to imply that in-line commenting itself is novel to the Discovery Stack model. Our intent was to emphasize the integration of in-line annotation with independent Quality and Impact assessments, standardized evaluation criteria, quantitative metrics, and a framework for dynamic post-publication evaluation. To clarify this distinction, we revised the Discussion to acknowledge prior implementations of in-line annotation during peer review and to more clearly describe how the Discovery Stack model builds upon these existing approaches.

I re-ran the code supplied at github to check the reproducibility of the work. Yes, every single line. It is well-documented and the results generated match what is in the manuscript.  It was interesting to see from this code-level review that the sample size in the Quality vs Impact Dispersion analyses (figures 5A to 5D) is on just 16 manuscripts, not 18. This is somewhat disclosed in the figure legend text for figure 5, although it would be nice to make it more consistent with the other figure legends to spell it out with "n=16".

**Response:** We thank the reviewer for taking the time to independently reproduce the analyses and for confirming that the supplied code generated the results reported in the manuscript. We appreciate the suggestion regarding clarity of the figure legend. To improve consistency with the other figure legends, we revised the legend for Figure 5 to explicitly state that the Quality versus Impact dispersion analyses were performed on n = 16 manuscripts.

**Reviewer #2:
[identifies himself as Stephen Curry]**

I am satisfied by the way that the authors have addressed the comments I raised in relation to their original submission.

One change to the revised manuscript does not pass muster. The claim on page 21 that 'a system like the one we tested would in the long run be at least as predictive of the long-term citation/value of a given piece of science as the journal impact factor' is not reasonable because the JIF - an average for the journal - is not a good predictor of scientific value of a the individual articles therein. This claim should be deleted since it adds nothing of merit.

**Response:** We thank the reviewer for the positive assessment of our revisions and are pleased that the previous concerns were satisfactorily addressed. We also appreciate the reviewer for highlighting this remaining issue. We agree that the statement was overly speculative and extended beyond what could be concluded from the present pilot study. Accordingly, we removed it from the manuscript. We also relocated the accompanying figure (**Fig S2** in the revised manuscript) to the Results section, where it now illustrates the observed relationship between reviewer-assessed Impact scores and JIF tiers rather than supporting speculation regarding long-term citation patterns or future predictive performance.

**Reviewer #3:
[identifies himself as Ludo Waltman]**

I consider the revised paper to be suitable for publication in the journal.

I still have a few minor comments:

"In fact, growing evidence suggests that the quality of published research inversely correlates with journal impact factor, which is evidenced by the higher retraction rates in journals with high impact factors (11, 14).": I wonder how strong this evidence is. Couldn't the inverse correlation be the result of articles in high impact factor journals receiving more attention than articles in low impact factor journals? This then increases the probability of the detection of errors in articles in high impact factor journals and, consequently, the probability of retraction of these articles.

**Response:** We thank the reviewer for this thoughtful observation. We agree that higher retraction rates among journals with higher JIFs may reflect greater visibility and scrutiny, rather than differences in the underlying quality of published research. Accordingly, we revised the Introduction to acknowledge this alternative interpretation and to avoid implying that retraction rates alone demonstrate an inverse relationship between research quality and JIF. Instead, we emphasize that JIF may not be a reliable proxy for the methodological quality or reliability of individual publications.

"The lack of significant correlation between Quality scores and journal impact factor is consistent with evidence that journal rank is a poor surrogate for scientific reliability (11).": I see the lack of a statistically significant correlation as a reflection of the small sample size. For a large enough sample size, it seems likely that the correlation will be statistically significant (but not necessarily substantively significant).

**Response:** We thank the reviewer for this important point and agree that the non-significant association between Quality scores and JIFs may reflect the modest sample size of this pilot. Accordingly, we revised the Results to state that the modest sample size may have limited our ability to detect a statistically significant association and clarify that these findings are consistent with previous evidence that JIF is a poor surrogate for scientific reliability.

"Journal impact factors are conceptually and statistically flawed (9-14).": Impact factors are causing lots of problems, but I don't agree they are statistically flawed. In my view, commonly used arguments about impact factors being statistically flawed are themselves statistically flawed. See [https://urldefense.com/v3/__https://doi.org/10.12688/f1000research.23418.2__;!!LQC6Cpwp!q6kGxYJ1KExfPeLl8xJXWQuULJ_P9guNyYsnAW9EqqWO0eycFYIw6aucHQrsT8VxSHVZAKQfeeoR_HVJ6Dt7teM$](https://urldefense.com/v3/__https:/doi.org/10.12688/f1000research.23418.2__;!!LQC6Cpwp!q6kGxYJ1KExfPeLl8xJXWQuULJ_P9guNyYsnAW9EqqWO0eycFYIw6aucHQrsT8VxSHVZAKQfeeoR_HVJ6Dt7teM$) .

**Response:** We thank the reviewer for this thoughtful comment and the reference. We recognize that the characterization of the Journal Impact Factor (JIF) as "statistically flawed" overstated a claim and is subject to debate. Accordingly, we revised the Discussion to remove the statement that JIFs are "statistically flawed" and instead describe their conceptual and practical limitations, including the highly skewed distribution of citations within journals, the use of the arithmetic mean, and the negotiability of the denominator. We also revised the surrounding text to emphasize that JIF is a poor proxy for the rigor and reliability of individual studies rather than making broader claims about the statistical validity of the metric itself.

Finally, I would like to note that I was slightly disappointed that the authors have not uploaded their revised article to bioRxiv. The version of the article on bioRxiv is outdated. As a result, I am unable to publish this review report online. I hope the authors will still update their article on bioRxiv.

**Response**: We thank the reviewer for this suggestion and appreciate the importance of maintaining an up-to-date preprint. This was an oversight and have uploaded the revised version of the manuscript to bioRxiv so that it reflects the current version under consideration and facilitates public discussion and posting of review reports.

Ludo Waltman
July 30, 2026

__________________________________________________
