# Supplementary material for: Discovery Stack Pilot demonstrates the feasibility and outcomes of a scientist-designed peer-review model that separates quality and impact
Source: PLoS Biol. 2026 Sep 8;24(9):e3003986. doi: 10.1371/journal.pbio.3003986 (PMC13614658; doi:10.1371/journal.pbio.3003986)
Supplement: Peer review history

# Editor report: Decision Letter 0

**DOI:** 10.1371/journal.pbio.3003986.r001

**Authors:** Roland Roberts

26 Nov 2025

Dear Dr Krummel,

Thank you for submitting your manuscript entitled "Discovery Stack Pilot: Feasibility and Outcomes of a Scientist-Designed Peer Review Model Separating Quality and Impact" for consideration as a Meta-Research Article by PLOS Biology. Please accept my apologies for the delayed incurred while I was travelling.

Your manuscript has now been evaluated by the PLOS Biology editorial staff, and I'm writing to let you know that we would like to send your submission out for external peer review.

However, before we can send your manuscript to reviewers, we need you to complete your submission by providing the metadata that is required for full assessment. To this end, please login to Editorial Manager where you will find the paper in the 'Submissions Needing Revisions' folder on your homepage. Please click 'Revise Submission' from the Action Links and complete all additional questions in the submission questionnaire.

Once your full submission is complete, your paper will undergo a series of checks in preparation for peer review. After your manuscript has passed the checks it will be sent out for review. To provide the metadata for your submission, please Login to Editorial Manager (https://www.editorialmanager.com/pbiology) within two working days, i.e. by Nov 28 2025 11:59PM.

If your manuscript has been previously peer-reviewed at another journal, PLOS Biology is willing to work with those reviews in order to avoid re-starting the process. Submission of the previous reviews is entirely optional and our ability to use them effectively will depend on the willingness of the previous journal to confirm the content of the reports and share the reviewer identities. Please note that we reserve the right to invite additional reviewers if we consider that additional/independent reviewers are needed, although we aim to avoid this as far as possible. In our experience, working with previous reviews does save time.

If you would like us to consider previous reviewer reports, please edit your cover letter to let us know and include the name of the journal where the work was previously considered and the manuscript ID it was given. In addition, please upload a response to the reviews as a 'Prior Peer Review' file type, which should include the reports in full and a point-by-point reply detailing how you have or plan to address the reviewers' concerns.

During the process of completing your manuscript submission, you will be invited to opt-in to posting your pre-review manuscript as a bioRxiv preprint. Visit http://journals.plos.org/plosbiology/s/preprints for full details. If you consent to posting your current manuscript as a preprint, please upload a single Preprint PDF.

Feel free to email us at plosbiology@plos.org if you have any queries relating to your submission.

Kind regards,

Roli Roberts

Roland Roberts, PhD

Senior Editor

PLOS Biology

rroberts@plos.org

# Peer review: Decision Letter 1

**DOI:** 10.1371/journal.pbio.3003986.r002

**Authors:** Roland Roberts

19 Mar 2026

Dear Dr Krummel,

Thank you for your patience while your manuscript "Discovery Stack Pilot: Feasibility and Outcomes of a Scientist-Designed Peer Review Model Separating Quality and Impact" was peer-reviewed at PLOS Biology. It has now been evaluated by the PLOS Biology editors and by three independent reviewers.

You'll see that reviewer #1 raises significant concerns about the robustness of your study, while reviewers #2 and #3 are more favourably disposed, seeing a valuable contribution even in this relatively preliminary state. During post-review cross-commenting, the latter reviewers maintained this position (while recognising rev #1's points), and in balance we share their opinion that there is value in considering your paper further. Naturally you should attempt to address reviewer #1's most pressing concerns (e.g. re weak statistical power and selection bias), whether by new analyses or flagging limitations, and in particular to acknowledge/emphasise the very preliminary nature of your findings (and it may well be that during the past 2-3 months you have accrued more data which may be able to strengthen your conclusions...).

In light of the reviews, which you will find at the end of this email, we would like to invite you to revise the work to thoroughly address the reviewers' reports.

Given the extent of revision needed, we cannot make a decision about publication until we have seen the revised manuscript and your response to the reviewers' comments. Your revised manuscript is likely to be sent for further evaluation by all or a subset of the reviewers.

In addition to these revisions, you will need to complete some formatting changes, which you will receive in a follow up email. A member of our team will be in touch with a set of requests shortly.

We expect to receive your revised manuscript within 3 months. Please email us (plosbiology@plos.org) if you have any questions or concerns, or would like to request an extension.

At this stage, your manuscript remains formally under active consideration at our journal; please notify us by email if you do not intend to submit a revision so that we may withdraw it.

**IMPORTANT - SUBMITTING YOUR REVISION**

Your revisions should address the specific points made by each reviewer. Please submit the following files along with your revised manuscript:

1. A 'Response to Reviewers' file - this should detail your responses to the editorial requests, present a point-by-point response to all of the reviewers' comments, and indicate the changes made to the manuscript.

*NOTE: In your point-by-point response to the reviewers, please provide the full context of each review. Do not selectively quote paragraphs or sentences to reply to. The entire set of reviewer comments should be present in full and each specific point should be responded to individually, point by point.

You should also cite any additional relevant literature that has been published since the original submission and mention any additional citations in your response.

2. In addition to a clean copy of the manuscript, please also upload a 'track-changes' version of your manuscript that specifies the edits made. This should be uploaded as a "Revised Article with Changes Highlighted" file type.

*Re-submission Checklist*

When you are ready to resubmit your revised manuscript, please refer to this re-submission checklist: https://plos.io/Biology_Checklist

To submit a revised version of your manuscript, please go to https://www.editorialmanager.com/pbiology/ and log in as an Author. Click the link labelled 'Submissions Needing Revision' where you will find your submission record.

Please make sure to read the following important policies and guidelines while preparing your revision:

*Published Peer Review*

Please note while forming your response, if your article is accepted, you may have the opportunity to make the peer review history publicly available. The record will include editor decision letters (with reviews) and your responses to reviewer comments. If eligible, we will contact you to opt in or out. Please see here for more details:

https://blogs.plos.org/plos/2019/05/plos-journals-now-open-for-published-peer-review/

*PLOS Data Policy*

Please note that as a condition of publication PLOS' data policy (http://journals.plos.org/plosbiology/s/data-availability) requires that you make available all data used to draw the conclusions arrived at in your manuscript. If you have not already done so, you must include any data used in your manuscript either in appropriate repositories, within the body of the manuscript, or as supporting information (N.B. this includes any numerical values that were used to generate graphs, histograms etc.). For an example see here: http://www.plosbiology.org/article/info%3Adoi%2F10.1371%2Fjournal.pbio.1001908#s5

*Blot and Gel Data Policy*

We require the original, uncropped and minimally adjusted images supporting all blot and gel results reported in an article's figures or Supporting Information files. We will require these files before a manuscript can be accepted so please prepare them now, if you have not already uploaded them. Please carefully read our guidelines for how to prepare and upload this data: https://journals.plos.org/plosbiology/s/figures#loc-blot-and-gel-reporting-requirements

*Protocols deposition*

To enhance the reproducibility of your results, we recommend that if applicable you deposit your laboratory protocols in protocols.io, where a protocol can be assigned its own identifier (DOI) such that it can be cited independently in the future. Additionally, PLOS ONE offers an option for publishing peer-reviewed Lab Protocol articles, which describe protocols hosted on protocols.io. Read more information on sharing protocols at https://plos.org/protocols?utm_medium=editorial-email&utm_source=authorletters&utm_campaign=protocols

Thank you again for your submission to our journal. We hope that our editorial process has been constructive thus far, and we welcome your feedback at any time. Please don't hesitate to contact us if you have any questions or comments.

Sincerely,

Roli Roberts

Roland Roberts, PhD

Senior Editor

PLOS Biology

rroberts@plos.org

------------------------------------

REVIEWERS' COMMENTS:

Reviewer #1:

[identifies himself as Ross Mounce]

Those who seek to critique the current academic publication system really ought to make sure that their work is robust, well designed, and well evidenced. In my opinion this manuscript falls very short in terms of scientific quality.

The experimental design of this manuscript is obviously flawed for many different reasons including:

A) insufficient sample size

B) underexplained highly subjective recruitment, making it prone to possible bias

C) duration: trying to analyse the experiment too early before all the results are known

D) lack of demographic transparency or stratification in the sample recruitment

Many of these experimental design issues could have been pointed out and corrected before any data was collected (and effort wasted?) if the researchers behind this study had submitted their experimental design as a registered report for peer review. I am thus also unsurprised to note that the authors appear to make no mention to or reference of registered reports in their manuscript. The authors might want to read a review article on registered reports: Chambers, C.D., Tzavella, L. The past, present and future of Registered Reports. Nat Hum Behav 6, 29-42 (2022). https://doi.org/10.1038/s41562-021-01193-7

I shall try to explain the perceived experimental design flaws in more detail below:

A) **Sample size**

The recruitment of merely 18 preprints, only 17 of which could be included in all analyses is bafflingly small and makes this research statistically underpowered assuming the effect sizes are small (e.g. if Cohen's d = 0.2). I can see that this is labelled as a "pilot" and thus can't be expected to be an industry-wide experiment but equally I'd ask what good is such a tiny and insufficiently described experiment? Did the authors do any kind of prospective power analysis before collecting data to see how many preprints they ought to recruit to get meaningful results?

The small overall sample size is further undermined by the fact that the sample was recruited in three different ways. On of the recruitment methods recruited just three preprints, so a sample size of just three for that subset. Does the recruitment method not have an impact? I think it does. What sample size for each of the recruitment methods would you need to prove me wrong?

What if I were to assert that two of the recruited preprints were highly atypical papers? This is why you need a larger N for the number of recruited preprints, to accommodate for any outliers that might be in the sample.

B) **Underexplained highly subjective recruitment**

Why were three different recruitment methods employed?

1)Editor emailed DSP-enrolled authors

2)Editor emailed BioRxiv authors

3)Selected from BioRxiv - authors did not enroll

In category 3 can we please have more detail on how and why three were "selected by the pilot's scientific advisory board and reviewed without author input". There were over 43,000 preprints posted to BioRxiv in 2024, why were the three selected arbitrarily chosen out of thousands of other possible choices?

I suggest a more rigorous experimental design would have been to use a random number generator to randomly select bioRxiv preprints for recruitment into this experiment. "Three additional bioRxiv manuscripts were selected by the scientific advisory board…" leaves this study open to accusations of cherry-picking and bias in how these three extra preprints were selected.

Another bias is in category 1 ("Editor emailed DSP-enrolled authors") this is textbook self-selection bias. These DSP-enrolled authors have already shown interest and engagement in the Discovery Stack Project and so these are included? Would it not be a stronger test of the discovery stack model to recruit authors/preprints who have not heard of or previously engaged with the discovery stack model? The inclusion of DSP-friendly or DSP-aware authors in the experiment invalidates some of the results such as on page 10:

"Moreover, 84.9% of authors and reviewers agreed that separating these dimensions led to more constructive and insightful evaluations than traditional reviews"

because of self-selection bias. You're sampling from people who like the idea and so it's unsurprising that these authors might agree with reviewers who have also agreed to test the discovery stack model. Homophily.

C) **Duration**

There's a remarkable degree of impatience displayed in this manuscript: "only six of 18 manuscripts had been accepted at the time of analysis". Why not simply wait a few more months before publishing an analysis so that the ultimate journal venue of publication can be analysed for >90% of the recruited preprints? Why rush to publish so prematurely? I think knowing the final publication venue for >90% of the recruited preprints would be valuable data to include in any serious analysis of this model.

D) ** lack of demographic transparency or stratification in the sample recruitment **

The 18 preprints that form the core of this work appear to have been anonymised with labels such as DSPM118 and DSPL178. Why the anonymisation? If these are preprints published online at BioRxiv then they are published and known. I don't see why they need to be anonymised. It decreases my trust in this research that I don't know the exact identity of the 18 preprints that were recruited.

As I think we all recognise, no two scientific preprints are the same - a preprint isn't a simple substitutable unit.

What are the demographic variations of each of these preprints?

i.e. How many authors are named on each preprint?

How many pages are each preprint?

How many different institutions are affiliated with each paper? E.g. (1-100+)

Which countries are the listed author institutions on each preprint?

You can't call the discovery stack model a success if it's only been piloted with US & UK based academics… There needs to be explicit sociopolitical stratification built into the experimental design of the testing.

Aside from the issues with the experiment design, I also take issue with the near total absence of the importance of software in the discussion of Quality.

The authors state: "Quality refers to the rigor and reproducibility of the data supporting a study's conclusions"

Is that it? Really? Data only?

What about the critical role of software? Software should really get a mention as being part and parcel of research quality. As has been said elsewhere "The future of research software is the future of research" [1] 7 out of 10 UK-based researchers agree with the statement that it's impossible to conduct research without software. [2]

[1] Chue Hong NP, Aragon S, Hettrick S, Jay C. The future of research software is the future of research. Patterns (N Y). 2025 Jul 11;6(7):101322. doi: 10.1016/j.patter.2025.101322. PMID: 40926964; PMCID: PMC12416084.

[2] Simon Hettrick (2014) https://www.software.ac.uk/blog/its-impossible-conduct-research-without-software-say-7-out-10-uk-researchers

I specifically also examined the Quality Assessment Checklist given in supplementary figure 4 - there was no mention of software or code here either. Reading the Quality Assessment Checklist I inferred that it was devised and/or written by wet lab biologists: "identify experiments not repeated a standard of three times" being quite an obvious tell in this regard. If there is a desire to expand the discovery stack model to further disciplines then specific items like this will need to be revised...

I note that the word "code" features in Supplementary Figure 3 panel B and panel C and a throwaway mention on page 21 of the manuscript as something that was actually suggested by survey participants (well done to those participants that mentioned it!): "Other suggestions included easier access to raw data, code, and protocols; moderation of comments and vetting of users and reviewers; and an initial quality screen before review."

The discovery stack model absolutely must be amended to include data and software/code (with parity) within the consideration of research Quality. Examining the quality of research with no regard to its underlying software/code is madness.

Poor code can completely invalidate research and thus code must be inspected for quality. See e.g. the famous Reinhart and Rogoff debacle in economics

Herndon, Thomas & Ash, Michael & Pollin, Robert. (2013). Does High Public Debt Consistently Stifle Economic Growth? A Critique of Reinhart and Rogoff. Cambridge Journal of Economics. 38. 257-279. 10.1093/cje/bet075

The unwise disregard of code is evident within these authors own manuscript. I note with displeasure that on page 33 of the manuscript being reviewed the authors write:

"Bootstrap resampling was performed using custom Python scripts to assess the SD, range, and IQR of Quality and Impact scores"

Where are these Python scripts? This reviewer would very much like to see these Python scripts before being able to decide on the quality of this manuscript. It would be good if the authors could share the code behind their analyses at a trusted data repository such as Zenodo or Dryad.

*For the sake of reproducibility, what version of the GraphPad Prism software did the authors use and what version of NumPy?

*Where is there a copy of the exact survey instrument deposited, detailing all the questions and possible answers that were distributed to the 93 individuals (page 33). This does not seem to be contained within any of the supplementary figures.

*Curiously the author feedback survey is said to have been completed by 11 of 18 authors (61%). Preprints in the fields of Immunology and Cancer Biology do not tend to be authored by just a single person. Surely the feedback survey should poll ALL the authors of each preprint in order to get the views of all the authors? I would thus expect the total number of authors to be something like more than 100 not just 18.

*I note the manuscript only cites 19 other sources. Considering the topic I find it a little bit insufficient. More comparison and acknowledgement needs to be made to both registered reports and the publish, review, curate (PRC) model as relevant alternatives (that share important similarities) to the discovery stack model. Aczel et al (2021) should be cited to highlight the financial and time cost of current peer review systems.

Aczel, B., Szaszi, B. & Holcombe, A.O. A billion-dollar donation: estimating the cost of researchers' time spent on peer review. Res Integr Peer Rev 6, 14 (2021). https://doi.org/10.1186/s41073-021-00118-2

*Lack of discussion of the relevance or not of Journal Impact Factor ™ numbers created and sold by Clarivate. The authors describe Journal Impact Factor numbers as merely "flawed". I think this is somewhat of an understatement.

See e.g.

Curry, S. (2012, August 13). Sick of Impact Factors. Reciprocal Space. https://doi.org/10.59350/xqrv5-7bv94

Brembs B, Button K, Munafò M. Deep impact: unintended consequences of journal rank. Front Hum Neurosci. 2013 Jun 24;7:291. doi: 10.3389/fnhum.2013.00291. PMID: 23805088; PMCID: PMC3690355.

Dimitrov JD, Kaveri SV, Bayry J. Metrics: journal's impact factor skewed by a single paper. Nature. 2010 Jul 8;466(7303):179. doi: 10.1038/466179b. PMID: 20613817.

*The lack of significant correlation of (submitted) Journal Impact Factor ™ numbers with the Quality assessment is a fascinating result and should be discussed with reference to Brembs work:

Brembs B. Prestigious Science Journals Struggle to Reach Even Average Reliability. Front Hum Neurosci. 2018 Feb 20;12:37. doi: 10.3389/fnhum.2018.00037. Erratum in: Front Hum Neurosci. 2018 Oct 09;12:376. doi: 10.3389/fnhum.2018.00376. PMID: 29515380; PMCID: PMC5826185.

I am delighted that the authors get a result yet again confirming that the research quality of individual papers has no significant correlation with (submitted) Journal Impact Factor (Figure 2E). That is a key finding that more scientists and policymakers should take on board. Although the low sample size again is a bit unfortunate… Will this hold up with a larger sample size?

Reviewer #2:

[identifies himself as Stephen Curry]

This is a very interesting manuscript reporting the results of a pilot study of a peer review model that separates the assessment of quality and impact and enables the used of in-line reviewer comments. Although the scale of the pilot is relatively small (18 manuscripts assessed; 162 quality and/or impact reviews obtained) and focused on just two biomedical subfields (cancer biology and immunology), it has been performed and reported very thoroughly - albeit a little too thoroughly in some repects, given the small numbers in the surveys that make up a large part of the analysis. That said, overall, it makes for a valuable contribution to the literature on peer review which, event at this late stage, remains something of a black box.

There are a number of weaknesses in the study that I think should be addressed before publication.

The authors do a good job of showing that reviewers are able to meaningfully distinguish quality from impact (p8-9), but I wanted to see a clearer analysis of the claimed support for standardized metrics (p10). Although the methods for arriving at quantitative scores for quality and impact are explained clearly in the Methods section (i.e. using separate questionnaires with Likert-Scale questions), I think a brief outline of the assessment process should be presented alongside the results. It strikes me that the reported support for standardized metrics could well also arise from the fact that the questionnaires provide clearly defined criteria for the quality and impact assessments and that it is this clarity of criteria (still very rare in normal practice) that appealed to authors, alongside the scoring mechanism for arriving at the metrics. In fact, I think the use of defined criteria is such an important feature of the review protocol at the heart of the study that Supplementary Figures 6 and 7 should be promoted to the main body of the manuscript, rather than being relegated to the supplementary information, where they will more easily be missed.

Given the very small number of authors surveyed in the study, I think the manuscript authors need to be much more circumspect about concluding that "Identify disclosure enhances perceived transparency" (p11-12). They also place emphasis on the observation that 4/10 authors "stated that identified reviews were more constructive than anonymous reviews", seemingly overlooking their finding that 6/10 authors found there was no difference or that reviewers were less constructive. I think the finding here is that the results are inconclusive! In any case it should be emphasised that findings based on such small numbers must be regarded as extremely preliminary.

In many places the survey results are quoted to 3 significant figures. Given the small number of participants involved (<100), I think that a maximum of 2 significant figures would be more appropriate. This would also have the advantage of making the manuscript more readable.

On p13-14 the section titled "Discovery Stack Platform Delivers a Better Experience than Traditional Review" discusses authors' and reviewers' perceptions of their new platform. When discussing the authors' responses, I think it would be worth considering the fact that the review process did not produce an accept/reject decision and discussing whether that might colour author perceptions.

At the top of p14 it is stated that "Most participants (75%) agreed that the platform fostered this mindset more effectively than traditional reviews". However, I would question whether the platform is solely responsible for this perception. In the Methods it is revealed that the briefing given to reviewers "emphasized the value of a peer-improvement mindset". At the very least, this instruction would appear to be a confounder of the analysis. The authors should remind the reader in presenting the results that this instruction was given and comment on its likely impact.

On p18-19 there is a section titled "Expedited Review Is Achievable with Reviewer Accountability and Backup Plans". The purpose of this section is not clear to me. The authors report review times for quality and impact assessments, but then claim that their pilot cannot be compared directly to traditional review. They then proceed to discuss the impact of single late reviews and the need for contingency plans. In the absence of an experiment designed to directly compare traditional review with the Discovery Stack model, I don't see that this section adds any great value to the manuscript.

The final two sections of the results (p20-21) probe the surveys for their interest in new publishing models and their views on desirable features. Again, given the small sample size, I don't think too much can be made of these results. Moreover, the answers to questions about using 'scientist-developed metrics to assess quality and impact' must be difficult to interpret if the form of the metrics is not discussed.

We are told also in the last section (p21) that 71.8% of respondents would prioritise "evolving impact metrics based on ongoing community input". Maybe so - and I agree that impact is very difficult to assess a priori - but how would such "community input" be gathered? In the absence of any realistic prospect for a mechanism, such aspirations come across as somewhat naïve. I think here and in the discussion the authors should discuss the practical obstacles to "dynamic impact metrics".

The Discussion section also raises the possibility that in time "a threshold for high-Quality papers may emerge" from the use of standardized metrics. Although the authors are careful to acknowledge the risks associated with metrics, they don't outline a process for re-scoring quality on their platform. As presented, it produces a quality score for the submitted, not the revised manuscript. Re-scoring would add a further burden on reviewers, as would any attempt to keep track of impact months or years after publication. In any case, does this add substantial value above and beyond the practice of open review (in which reviews are published)? This should be discussed.

The discussion of the "broad dissatisfaction with current publishing norms" seems to me to add nothing new to our understanding of present woes and could be truncated. Happy to be corrected if I have missed something.

Overall, I have the impression that the numerous and detailed analyses presented in this paper end up muddying or masking what I think are its most important messages. These are the demonstrable value of a review protocol that separates quality from impact, AND assesses these using multiple, clearly defined criteria scored in a standardised way. The second important innovation is the utility of introducing in-line reviewer comments. I think some of the weaker or less important results sections could be truncated (or relegated to the supplementary information) and this would allow the central findings to come through more strongly.

It would also help to have a stronger focus on the practicalities, given the well-known pressures on the peer review system. A protocol that needs 2-3 quality reviewers and 5-6 impact reviewers doesn't seem likely to take off. Would it be more realistic to have fewer reviewers, who both score quality and impact (using the different criteria) and down-weight the impact score given the greater uncertainty associated with that assessment? A sharper consideration of the practicalities would in my view enhance the quality of the present manuscript and augment the probability that it will have a significant impact!

Reviewer #3:

[identifies himself as Ludo Waltman]

My review report is available online: https://prereview.org/reviews/18915960.

Ludo Waltman

March 8, 2026

FLAT TEXT VERSION OF THAT REVIEW:

This paper presents the outcomes of a pilot study of a new approach to peer review of research articles. In this new approach, the quality and impact of an article are evaluated separately. I found the paper highly interesting to read. As pointed out by the authors, established approaches to peer review are facing major challenges. There is a strong need for studying alternative approaches to peer review.

My comments on the paper are fairly minor.

My most important comment is that the idea of separating the evaluation of quality and impact is not new. The authors should acknowledge existing approaches based on this idea. Let me highlight two approaches.

First, an increasing number of journals are using a so-called soundness-only approach to peer review. These journals ask reviewers to evaluate the soundness (i.e., quality) of an article and to refrain from evaluating the impact of an article. Soundness-only peer review was pioneered by PLOS One. Nowadays it is also used by various other journals, such as Scientific Reports. There is also literature studying soundness only peer review. See https://doi.org/10.1108/JD-06-2017-0092.

Second, the journal eLife uses a publish-review-curate model in which an explicit distinction is made between the strength of evidence (i.e., quality) and the significance of the findings (i.e., impact). Strength of evidence and significance of the findings are each assessed on a five- or six-point scale. There seem to be close connections between the eLife model and the Discovery Stack model proposed by the authors. The authors should discuss these connections.

A minor comment relates to the way in which the authors use the word ‘published’. The authors for instance state: “At the time of analysis, only six of 18 manuscripts had been published. The mean from submission to publication for the published manuscripts was 231 days.” This is confusing. Articles can be published (i.e., be made publicly available) not only in journals but also on other platforms, such as preprint servers. In this case, all 18 manuscripts have already been published on a preprint server. Therefore, instead of “only six of 18 manuscripts had been published”, the authors should say “only six of 18 manuscripts had been published in a journal”. Likewise, “from submission to publication” should be “from submission to publication in a journal”.

Finally, I was unable to find the data underlying the analyses presented in the paper. In the interest of transparency and reproducibility, publishing the data is crucial.

Competing interests

The author declares that they have no competing interests.

Use of Artificial Intelligence (AI)

The author declares that they did not use generative AI to come up with new ideas for their review.

# Author comment: Author response to Decision Letter 2

**DOI:** 10.1371/journal.pbio.3003986.r003

26 Jun 2026

Attachment Submitted filename: Response_to_Reviewers_MFK.docx

# Peer review: Decision Letter 2

**DOI:** 10.1371/journal.pbio.3003986.r004

**Authors:** Roland Roberts

3 Aug 2026

Dear Max,

Thank you for your patience while we considered your revised manuscript "Discovery Stack Pilot: Feasibility and Outcomes of a Scientist-Designed Peer Review Model Separating Quality and Impact" for publication as a Meta-Research Article at PLOS Biology. This revised version of your manuscript has been evaluated by the PLOS Biology editors, the Academic Editor and the original reviewers.

Based on the reviews, we are likely to accept this manuscript for publication, provided you satisfactorily address the remaining points raised by the reviewers, and the following data and other policy-related requests.

IMPORTANT - please attend to the following:

a) We need your Title to contain an active verb and to avoid a colon (these tend to result in title truncation by some aggregators). We suggest changing it to: "A pilot study reveals the feasibility and outcomes of Discovery Stack, a scientist-designed peer peview model that separates quality and impact"

b) Please attend to the remaining comments from the reviewers.

c) Please confirm that this process, which involved human subjects in a trial peer review process, did not require ethical approval.

d) We note that you specify one author as receiving "specific" support for this project. Please also declare any non-specific support which was used for this project.

e) Please address my Data Policy requests below; specifically, we need you to supply the numerical values underlying Figs 1BC, 4ABCDEFGHIJ, 5ABCD, 6ABCD, 7ABC, 8ABCD, 9ABCDEFGHIJ, 10AB, S1CDEF, S2AB, S3ABCDEFGHIJKLM, S4ABCDEFGH, S5ABC, S7AB, either as a supplementary data file or as a permanent DOI’d deposition.

f) Please cite the location of the data clearly in all relevant main and supplementary Figure legends, e.g. “The data underlying this Figure can be found in S1 Data” or “The data underlying this Figure can be found in https://zenodo.org/records/XXXXXXXX; ; also update your Data Availability Statement accordingly.

g) Please make any custom code available, either as a supplementary file or as part of your data deposition.

h) Please include the URLs of your funders in the Financial Disclosure statement.

As you address these items, please take this last chance to review your reference list to ensure that it is complete and correct. If you have cited papers that have been retracted, please include the rationale for doing so in the manuscript text, or remove these references and replace them with relevant current references. Any changes to the reference list should be mentioned in the cover letter that accompanies your revised manuscript.

In addition to these revisions, you may need to complete some formatting changes, which you will receive in a follow up email. A member of our team will be in touch with a set of requests shortly. If you do not receive a separate email within a few days, please assume that checks have been completed, and no additional changes are required.

We expect to receive your revised manuscript within two weeks.

To submit your revision, please go to https://www.editorialmanager.com/pbiology/ and log in as an Author. Click the link labelled 'Submissions Needing Revision' to find your submission record. Your revised submission must include the following:

- a cover letter that should detail your responses to any editorial requests, if applicable, and whether changes have been made to the reference list

- a Response to Reviewers file that provides a detailed response to the reviewers' comments (if applicable, if not applicable please do not delete your existing 'Response to Reviewers' file.)

- a track-changes file indicating any changes that you have made to the manuscript.

NOTE: If Supporting Information files are included with your article, note that these are not copyedited and will be published as they are submitted. Please ensure that these files are legible and of high quality (at least 300 dpi) in an easily accessible file format. For this reason, please be aware that any references listed in an SI file will not be indexed. For more information, see our Supporting Information guidelines:

https://journals.plos.org/plosbiology/s/supporting-information

*Published Peer Review History*

Please note that you may have the opportunity to make the peer review history publicly available. The record will include editor decision letters (with reviews) and your responses to reviewer comments. If eligible, we will contact you to opt in or out. Please see here for more details:

https://plos.org/published-peer-review-history/

*Press*

Should you, your institution's press office or the journal office choose to press release your paper, please ensure you have opted out of Early Article Posting on the submission form. We ask that you notify us as soon as possible if you or your institution is planning to press release the article.

*Protocols deposition*

To enhance the reproducibility of your results, we recommend that if applicable you deposit your laboratory protocols in protocols.io, where a protocol can be assigned its own identifier (DOI) such that it can be cited independently in the future. Additionally, PLOS ONE offers an option for publishing peer-reviewed Lab Protocol articles, which describe protocols hosted on protocols.io. Read more information on sharing protocols at https://plos.org/protocols?utm_medium=editorial-email&utm_source=authorletters&utm_campaign=protocols

Please do not hesitate to contact me should you have any questions.

Sincerely,

Roli

Roland Roberts, PhD

Senior Editor

rroberts@plos.org

PLOS Biology

------------------------------------------------------------------------

ETHICS STATEMENT:

-- Please include information about the form of consent (written/oral) given for research involving human participants. All research involving human participants must have been approved by the authors' Institutional Review Board (IRB) or an equivalent committee, and must have been conducted according to the principles expressed in the Declaration of Helsinki.

------------------------------------------------------------------------

DATA POLICY:

You may be aware of the PLOS Data Policy, which requires that all data be made available without restriction: http://journals.plos.org/plosbiology/s/data-availability. For more information, please also see this editorial: http://dx.doi.org/10.1371/journal.pbio.1001797

Note that we do not require all raw data. Rather, we ask that all individual quantitative observations that underlie the data summarized in the figures and results of your paper be made available in one of the following forms:

1) Supplementary files (e.g., excel). Please ensure that all data files are uploaded as 'Supporting Information' and are invariably referred to (in the manuscript, figure legends, and the Description field when uploading your files) using the following format verbatim: S1 Data, S2 Data, etc. Multiple panels of a single or even several figures can be included as multiple sheets in one excel file that is saved using exactly the following convention: S1_Data.xlsx (using an underscore).

2) Deposition in a publicly available repository. Please also provide the accession code or a reviewer link so that we may view your data before publication.

Regardless of the method selected, please ensure that you provide the individual numerical values that underlie the summary data displayed in the following figure panels as they are essential for readers to assess your analysis and to reproduce it: Figs 1BC, 4ABCDEFGHIJ, 5ABCD, 6ABCD, 7ABC, 8ABCD, 9ABCDEFGHIJ, 10AB, S1CDEF, S2AB, S3ABCDEFGHIJKLM, S4ABCDEFGH, S5ABC, S7AB. NOTE: the numerical data provided should include all replicates AND the way in which the plotted mean and errors were derived (it should not present only the mean/average values).

IMPORTANT: Please also ensure that figure legends in your manuscript include information on where the underlying data can be found, and ensure your supplemental data file/s has a legend.

Please ensure that your Data Statement in the submission system accurately describes where your data can be found.

------------------------------------------------------------------------

CODE POLICY

Per journal policy, if you have generated any custom code during the course of this investigation, please make it available without restrictions. Please ensure that the code is sufficiently well documented and reusable, and that your Data Statement in the Editorial Manager submission system accurately describes where your code can be found. More information on our Code Policy, what and how to share can be found here: https://journals.plos.org/plosbiology/s/code-availability

Please note that we cannot accept sole deposition of code in GitHub, as this could be changed after publication. However, you can archive this version of your publicly available GitHub code to Zenodo. Once you do this, it will generate a DOI number, which you will need to provide in the Data Accessibility Statement (you are welcome to also provide the GitHub access information). See the process for doing this here: https://docs.github.com/en/repositories/archiving-a-github-repository/referencing-and-citing-content

------------------------------------------------------------------------

DATA NOT SHOWN?

- Please note that per journal policy, we do not allow the mention of "data not shown", "personal communication", "manuscript in preparation" or other references to data that is not publicly available or contained within this manuscript. Please either remove mention of these data or provide figures presenting the results and the data underlying the figure(s).

------------------------------------------------------------------------

REVIEWERS' COMMENTS:

Reviewer #1:

[identifies himself as Ross Mounce]

Thank you for the extensive and thoughtful revision. I appreciate the care with which you have engaged with the reviewers' comments and the substantial effort invested in clarifying the study design, expanding the discussion, improving transparency, and making the underlying data and code available.

Having read both the original review reports and your detailed responses, I believe the manuscript has improved considerably. In particular, the additions regarding recruitment, the explicit framing of the work as a feasibility pilot, the new analyses addressing familiarity bias, the provision of code and survey instruments, and the expanded discussion of related initiatives (e.g., soundness-only review, publish-review-curate approaches, and Registered Reports) all strengthen the manuscript and address many of the concerns raised previously.

That said, I remain somewhat cautious about the strength of several conclusions and would encourage further moderation of the manuscript's framing in a few areas.

**1. Pilot framing and generalizability | Overclaiming**

The revised manuscript does a much better job of describing itself as a feasibility study, and I commend the authors for explicitly acknowledging recruitment challenges, selection effects, and the limited size of the cohort. However, I still feel that some passages occasionally drift from "evidence of feasibility" toward broader claims regarding the supposed superiority or future success of the model.

The central contribution of this paper is not that the Discovery Stack model has been proven superior to existing peer review systems. Rather, it is that the authors have demonstrated the feasibility of recruiting 18 independent co-authors of preprints into implementing a new review process that separates assessments of Quality and Impact, incorporates structured criteria, and uses in-line commenting. The manuscript is strongest when it stays focused on these achievements.

For instance:

*the final sentence of the Discussion, the claim that the system "is ready for expansion and adoption" goes beyond what a pilot of 18 manuscripts can demonstrate. The study shows feasibility, not readiness for broad deployment.

*Quote from the revised manuscript: "In future implementations of the platform, a threshold for high-Quality papers may emerge, above which manuscripts would be designated as rigorous and trustworthy."

oThis is an interesting hypothesis, but it implies a level of validation that has not yet occurred. The pilot demonstrates that reviewers can generate Quality scores; it does not yet establish that those scores are sufficiently calibrated, reproducible, or predictive to support a threshold denoting trustworthy science.

*Quote from new text in the revised manuscript: "More examples would need to be produced but we consider it likely that a system like the one we tested would in the long run be at least as predictive of the long-term citation/value of a given piece of science as the journal impact factor."

oThis is tremendously speculative about the Discovery Stack model. I think it also manages to misrepresent the science of studies about Journal Impact Factor and its relation to the citedness of individual papers. Journal Impact Factor is a poor predictor of the citedness of individual papers within a journal. As shown by: Seglen, P.O. (1997). "Why the impact factor of journals should not be used for evaluating research." BMJ 314: 498-502. Zhang, L., Rousseau, R., & Sivertsen, G. (2017). "Science deserves to be judged by its contents, not by its wrapping." PLOS ONE 12:e0174205. Larivière, V. et al. (2016). "A simple proposal for the publication of journal citation distributions." and others...

**2. Recruitment bias remains an important limitation**

I appreciate the additional analyses comparing previously engaged participants and newly recruited participants. The finding that responses were broadly similar is useful and reassuring. Nevertheless, I do not think these analyses entirely eliminate concerns about participation bias.

The manuscript now documents that reviewer acceptance rates were dramatically higher among individuals already familiar with the project or contacted through existing networks. Indeed, the study reveals that personal outreach and prior engagement were the dominant determinants of participation.

To me, this is an interesting finding. It suggests that enthusiasm for alternative peer-review systems may not yet extend broadly across the wider research community and that adoption barriers remain substantial.

In the response to the reviewer comments, the authors disclose that only a single-author of each multi-author enrolled manuscript responded to the surveys. This masks variation in the role of each author in multi-author teams and how that maps to responses each author might give to the survey questions. What if the survey-responding author was a first-author, what if the survey-responding author was the PI on the grant and/or the last-author, what if the survey-responding author was a middle author amongst twenty other middle authors, who did the some of the pipetting work (minor involvement). It seems odd to treat all authors on a preprint as "the same", when particularly in terms of power, influence, and positionality towards change in scholarly communication, there are very important differences between researchers in different stages of their careers. This was not controlled for in the experimental design. Unfortunate.

**3. dynamic Impact metrics**

The revised discussion provides a much clearer explanation of how dynamic Impact metrics might work in practice. I found this addition helpful.

That said, implementation remains speculative. Citation counts, altmetrics, expert evaluations, downloads, policy citations, and social engagement all have known limitations and biases. I would encourage the authors to make clear that the proposed framework remains aspirational and untested rather than implying that an accepted methodology already exists.

### Overall assessment

My overall view is considerably more positive than in the previous round.

While I remain unconvinced that this study provides strong evidence for the effectiveness of the Discovery Stack model at scale, I do believe it now provides useful evidence that 18 anonymised preprints have undergone a Discovery Stack paid-review process. The manuscript is more transparent-ish, more balanced, better contextualized within the broader literature, and substantially more reproducible than the version originally reviewed.

However, one should not overlook the impact of paying peer-reviewers on the assessment of the model feasibility ($30 for each Quality review, $20 for each Impact review). Was it only feasible because you were offering direct financial payment? Would this level of payment be feasible if scaled up? Who exactly would provide the funding to pay for the payments to peer-reviewers and would they be happy to pay these extra fees, at a time when the United States Office of Management and Budget is proposing to stop paying from federal funds for any publication-related costs?[1] I'm not at all surprised that many happy volunteers were found to pilot this model given that payment was offered. Ker-ching! People like to earn more money. The understandable desire from an individual to earn more money may perhaps mask actual enthusiasm for the scholarly benefit(s) or disbenefit(s) of the model?

[1] https://sparcopen.org/our-work/2026-proposed-2cfr200-updates-faqs/ & https://www.federalregister.gov/documents/2026/05/29/2026-10817/regulation-for-federal-financial-assistance

Sidenotes:

A stylistic point. I would advise against lower-casing mentions of Clarivate's proprietary Journal Impact Factor ™ (JIF) numbers. Lower-casing gives the false impression that JIF numbers are somehow transparent, reproducible, or sensibly calculated. They are not. They are proprietary, negotiable, statistically-illiterate numbers which only Clarivate has the right to assign to journals as their official (Clarivate-awarded) Journal Impact Factor™ . References throughout to 'journal impact factor' or, even worse 'impact factor' [failing to mention the word journal, which neglects to explicitly inform that it is a journal-level number] may mislead readers into thinking that these are generic numbers created following some kind of rational and statistically-sound method - they are NOT!

Prior examples of using in-line commenting for peer-review of preprints include one by myself in 2021 https://greenelab.github.io/annorxiver_manuscript/ (see the Hypothesis logo in the top-right corner). This was journal-organized peer-review. The journal which organized it was PLOS Biology (!). The journal eventually published it here: https://journals.plos.org/plosbiology/article?id=10.1371/journal.pbio.3001470

These in-line Hypothes.is comments were incorporated into and extensively referenced in the submitted first-round peer review report https://journals.plos.org/plosbiology/article/peerReview?id=10.1371/journal.pbio.3001470

I'm sure there are many other examples, either more systematically implemented at a journal, or at an earlier date than 2021, such as at the American Geophysical Union (Hypothes.is and the Geophysical Electronic Manuscript Submission [GEMS] in editorial review) and JMIR Publications (Hypothes.is and preprints). The point is that in-line commenting for peer-review of preprints during journal-organized peer review is not completely new to the Discovery Stack model. I just wanted to make that clear.

I re-ran the code supplied at github to check the reproducibility of the work. Yes, every single line. It is well-documented and the results generated match what is in the manuscript. It was interesting to see from this code-level review that the sample size in the Quality vs Impact Dispersion analyses (figures 5A to 5D) is on just 16 manuscripts, not 18. This is somewhat disclosed in the figure legend text for figure 5, although it would be nice to make it more consistent with the other figure legends to spell it out with "n=16".

Reviewer #2:

[identifies himself as Stephen Curry]

I am satisfied by the way that the authors have addressed the comments I raised in relation to their original submission.

One change to the revised manuscript does not pass muster. The claim on page 21 that 'a system like the one we tested would in the long run be at least as predictive of the long-term citation/value of a given piece of science as the journal impact factor' is not reasonable because the JIF - an average for the journal - is not a good predictor of scientific value of a the individual articles therein. This claim should be deleted since it adds nothing of merit.

Reviewer #3:

[identifies himself as Ludo Waltman]

I consider the revised paper to be suitable for publication in the journal.

I still have a few minor comments:

"In fact, growing evidence suggests that the quality of published research inversely correlates with journal impact factor, which is evidenced by the higher retraction rates in journals with high impact factors (11, 14).": I wonder how strong this evidence is. Couldn't the inverse correlation be the result of articles in high impact factor journals receiving more attention than articles in low impact factor journals? This then increases the probability of the detection of errors in articles in high impact factor journals and, consequently, the probability of retraction of these articles.

"The lack of significant correlation between Quality scores and journal impact factor is consistent with evidence that journal rank is a poor surrogate for scientific reliability (11).": I see the lack of a statistically significant correlation as a reflection of the small sample size. For a large enough sample size, it seems likely that the correlation will be statistically significant (but not necessarily substantively significant).

"Journal impact factors are conceptually and statistically flawed (9-14).": Impact factors are causing lots of problems, but I don't agree they are statistically flawed. In my view, commonly used arguments about impact factors being statistically flawed are themselves statistically flawed. See https://doi.org/10.12688/f1000research.23418.2.

Finally, I would like to note that I was slightly disappointed that the authors have not uploaded their revised article to bioRxiv. The version of the article on bioRxiv is outdated. As a result, I am unable to publish this review report online. I hope the authors will still update their article on bioRxiv.

Ludo Waltman

July 30, 2026

# Author comment: Author response to Decision Letter 3

**DOI:** 10.1371/journal.pbio.3003986.r005

19 Aug 2026

Attachment Submitted filename: Response_to_Reviewers2nd.docx

# Editor report: Decision Letter 3

**DOI:** 10.1371/journal.pbio.3003986.r006

**Authors:** Roland Roberts

20 Aug 2026

Dear Max,

Thank you for the submission of your revised Meta-Research Article "Discovery Stack Pilot demonstrates the feasibility and outcomes of a scientist-designed peer-review model that separates quality and impact" for publication in PLOS Biology. On behalf of my colleagues and the Academic Editor, Stephen Curry, I'm pleased to say that we can in principle accept your manuscript for publication, provided you address any remaining formatting and reporting issues. These will be detailed in an email you should receive within 2-3 business days from our colleagues in the journal operations team; no action is required from you until then. Please note that we will not be able to formally accept your manuscript and schedule it for publication until you have completed any requested changes.

IMPORTANT:

a) You'll see that I've taken the liberty of changing your Title into sentence case, which is journal style; I did this to ensure that "Discovery Stack Pilot" will be capitalised correctly by our Production team.

b) I also took the liberty of including in your "competing interests statement" the fact that your co-author Ken Cadwell is a member of our editorial board. Your statement now reads, “I have read the journal’s policy and the authors of this manuscript have the following competing interests: KC is a member of PLOS Biology’s Editorial Board. The other authors declare that no competing interests exist."

c) I've asked my colleagues to include the following further request alongside their own: "Many thanks for depositing your code in Github. However, because Github depositions can be readily changed or deleted, please make a permanent DOI’d copy (e.g. in Zenodo) and provide this URL."

Please take a minute to log into Editorial Manager at http://www.editorialmanager.com/pbiology/, click the "Update My Information" link at the top of the page, and update your user information to ensure an efficient production process.

PRESS: We frequently collaborate with press offices. If your institution or institutions have a press office, please notify them about your upcoming paper at this point, to enable them to help maximise its impact. If the press office is planning to promote your findings, we would be grateful if they could coordinate with biologypress@plos.org. If you have previously opted in to the early version process, we ask that you notify us immediately of any press plans so that we may opt out on your behalf.

We also ask that you take this opportunity to read our Embargo Policy regarding the discussion, promotion and media coverage of work that is yet to be published by PLOS. As your manuscript is not yet published, it is bound by the conditions of our Embargo Policy. Please be aware that this policy is in place both to ensure that any press coverage of your article is fully substantiated and to provide a direct link between such coverage and the published work. For full details of our Embargo Policy, please visit http://www.plos.org/about/media-inquiries/embargo-policy/.

Thank you again for choosing PLOS Biology for publication and supporting Open Access publishing. We look forward to publishing your study.

Sincerely,

Roli

Roland G Roberts, PhD, PhD

Senior Editor

PLOS Biology

rroberts@plos.org
